# Agricultural Management Affects the Active Rhizosphere Bacterial Community Composition and Nitrification

**DOI:** 10.1128/mSystems.00651-21

**Published:** 2021-09-28

**Authors:** Guillaume Bay, Conard Lee, Chiliang Chen, Navreet K. Mahal, Michael J. Castellano, Kirsten S. Hofmockel, Larry J. Halverson

**Affiliations:** a Department of Plant Pathology and Microbiology, Iowa State University, Ames, Iowa, USA; b Department of Ecology, Evolution and Organismal Biology, Iowa State University, Ames, Iowa, USA; c Department of Agronomy, Iowa State University, Ames, Iowa, USA; d Interdepartmental Microbiology Graduate Program, Iowa State University, Ames, Iowa, USA; e Earth and Biological Sciences Directorate, Pacific Northwest National Laboratory, Richland, Washington, USA; f Affiliate Member, Ames National Laboratory, Ames, Iowa, USA; Lawrence Berkeley National Laboratory

**Keywords:** agricultural management, nitrification, rhizosphere-inhabiting microbes

## Abstract

Cropping system diversity provides yield benefits that may result from shifts in the composition of root-associated bacterial and fungal communities, which either enhance nutrient availability or limit nutrient loss. We investigated whether temporal diversity of annual cropping systems (four versus two crops in rotation) influences the composition and metabolic activities of root-associated microbial communities in maize at a developmental stage when the peak rate of nitrogen uptake occurs. We monitored total (DNA-based) and potentially active (RNA-based) bacterial communities and total (DNA-based) fungal communities in the soil, rhizosphere, and endosphere. Cropping system diversity strongly influenced the composition of the soil microbial communities, which influenced the recruitment of the resident microbial communities and, in particular, the potentially active rhizosphere and endosphere bacterial communities. The diversified cropping system rhizosphere recruited a more diverse bacterial community (species richness), even though there was little difference in soil species richness between the two cropping systems. In contrast, fungal species richness was greater in the conventional rhizosphere, which was enriched in fungal pathogens; the diversified rhizosphere, however, was enriched in *Glomeromycetes*. While cropping system influenced endosphere community composition, greater correspondence between DNA- and RNA-based profiles suggests a higher representation of active bacterial populations. Cropping system diversity influenced the composition of ammonia oxidizers, which coincided with diminished potential nitrification activity and gross nitrate production rates, particularly in the rhizosphere. The results of our study suggest that diversified cropping systems shift the composition of the rhizosphere’s active bacterial and total fungal communities, resulting in tighter coupling between plants and microbial processes that influence nitrogen acquisition and retention.

**IMPORTANCE** Crops in simplified, low-diversity agroecosystems assimilate only a fraction of the inorganic nitrogen (N) fertilizer inputs. Much of this N fertilizer is lost to the environment as N oxides, which degrade water quality and contribute to climate change and loss of biodiversity. Ecologically inspired management may facilitate mutualistic interactions between plant roots and microbes to liberate nutrients when plants need them, while also decreasing nutrient loss and pathogen pressure. In this study, we investigate the effects of a conventional (2-year rotation, inorganic fertilization) and a diversified (4-year rotation, manure amendments) cropping system on the assembly of bacterial and fungal root-associated communities, at a maize developmental stage when nitrogen demand is beginning to increase. Our results indicate that agricultural management influences the recruitment of root-associated microbial communities and that diversified cropping systems have lower rates of nitrification (particularly in the rhizosphere), thereby reducing the potential for loss of nitrate from these systems.

## INTRODUCTION

Global maize (Zea mays L.) production (tons/year) exceeds all other crops (1). Over the last 50 years, increasingly available synthetic nitrogen (N) fertilizers and pesticides have allowed farmers to reduce or eliminate annual crop rotations, such that maize is grown continuously or in rotation with only one additional crop. However, crops in these simplified, low-diversity cropping systems assimilate only a fraction of the N from fertilizers (10 to 50%) (2) and may have lost mutualistic interactions with soil microbes that enhance crop N-use efficiency (3). Thus, there is increased attention toward developing economically viable and environmentally friendly agroecosystems that reduce the need for synthetic N fertilizer and herbicides by increasing crop rotation diversity and integrating livestock (4–6). Ecologically inspired management may facilitate mutualistic interactions between plant roots and microbes, increasing the depolymerization of soil organic matter (7–9) to liberate nutrients, decrease pathogen pressure (10), stimulate plant growth (11), and/or facilitate stress tolerance (12).

Plants provide a suitable environment for the soil microbiome by producing carbon (C)-rich rhizodeposits to stimulate microbial metabolic activity near the root (13–15). The root-associated microbiome shifts as the plant develops, likely a consequence of changes in rhizodeposition (14–17). For example, expression of N metabolism genes in the Arabidopsis thaliana rhizosphere microbiome changes with shifts in root exudation (16). Consequently, root exudates, which can diffuse up to 5 mm from the root surface, appear to shape the rhizosphere microbiome (15, 16, 18). Bacteria residing at the root surface are influenced by plant roots and the soil they interact with, and they may play a particularly important role in plant-microbiome-mediated nutrient uptake due to the advantage microbes have over plants for recently mobilized N (19). Moreover, rhizosphere microbial communities are further influenced by agricultural management (20–22), resulting in unique interactions between the effects of management on the microbial seed bank and the influence of plant rhizodeposition on microbiome assembly and function.

The influence of crop rotation diversity on soil and rhizosphere metabolic activity and microbiome assembly has been examined at the Marsden Long-Term Cropping System Experiment in the Midwest USA Corn-belt. The diversified system (4-year rotation with a reduction in tillage, pesticides, and inorganic fertilization) is equally productive as the conventional system (2-year rotation with inorganic fertilization) (4), and it has lower environmental N losses (23). It also supports higher microbial biomass, greater net production of bioavailable N from soil organic matter, and potentially mineralizable N (24–26). However, gross rates of ammonia production in the bulk soil (i.e., root-free) did not differ substantially between cropping systems, suggesting that plant-microbe interactions, rather than bulk plant-available soil N production, play an unspecified role in boosting yields in the diversified system (24). Following up on these observations, we showed that management influenced the assembly of prokaryotic and fungal communities near and on the root as the plant develops (27). Interestingly, bulk soil and rhizosphere prokaryotic communities in the conventional system were significantly different from each other only at select plant developmental stages (V11 and R2), suggesting there was a transient rhizosphere effect. This could be a consequence of differential exudation by roots, analogous to how soil type and plant development influence the rhizosphere effect (11, 28). Using arbuscular mycorrhizal fungi (AMF)-proficient and -deficient corn, we showed that AMF aid plants in nitrate uptake but have little influence on the growth of ammonia oxidizers, regardless of cropping system (29).

Here we explore the following questions about the effects of diversified cropping systems on root microbiomes. (i) Does diversification influence the plant roots’ selection of the rhizosphere and endosphere microbiome? (ii) Does diversification impact rhizosphere priming and does this, in turn, influence the metabolic properties of the root-associated microbiome? (iii) If so, does this shift in metabolic capabilities influence N-cycling processes? We hypothesize that rhizosphere priming (18, 30, 31) is likely shaped by agricultural management effects on the microbial seed bank, resulting in shifts in community structure and their metabolic potential, and that these differences are greatest during periods of high plant nutrient demand (e.g., N). As a consequence of greater coupling between roots and microbes, we further predict that nitrification is lower in the rhizosphere than in the bulk soil of diversified agricultural systems. To test this hypothesis, we assessed the prokaryotic and fungal communities of the maize root-associated microbiome (rhizosphere and endosphere) and soil (no living root influence) in rhizotrons filled with soil from the conventional and diversified cropping system plots of our previous work (29–32). Plants were grown to the V4/V5 developmental stage, i.e., when the rate of maize nutrient uptake becomes greatest (32). Moreover, we complemented our assessment of the resident prokaryotic community (DNA-based) with the potentially metabolically active (RNA-based) prokaryotic community (33); this would provide insight into whether management influences rhizosphere priming, also because bacteria in close physical proximity of a root (e.g., rhizoplane) exhibit a high degree of selectivity in terms of residents and potentially active populations (34).

## RESULTS

### Diversified cropping systems change the root architecture of maize.

Roots of plants grown in soil from the diversified system exhibited a finer, more ramified architecture than those grown in soil from the conventional system (Table 1). Moreover, root/shoot ratios indicated that diversified cropping systems led to an increased allocation of plant biomass to roots. As reported previously for this site (4), we also observed that soil from the diversified cropping system had substantially lower nitrate pools (see Table S1 in the supplemental material), which may have stimulated greater root development in these soils.

**TABLE 1:** Maize root architectural features

Root trait	Mean ± SE		*P* value^*a*^
	Conventional	Diversified	
Total no. of roots	102 ± 13	169 ± 17	0.01
Length of primary root (cm)	11.2 ± 0.7	14.9 ± 1.5	0.09
Vol of primary root (cm^3^)	1350 ± 188	736 ± 112	0.03
Total surface area of roots (cm^2^)	23.1 ± 2.4	29.4 ± 1.7	0.04
Maximum depth of root system (cm)	19.1 ± 1.8	26.4 ± 3.2	0.07
Maximum horizontal width of roots (cm)	7.5 ± 0.7	10.3 ± 1.1	0.06
Root dry wt (g)	0.25 ± 0.03	0.19 ± 0.02	0.08
Root/shoot ratio (g/g)	0.11 ± 0.01	0.18 ± 0.02	0.01

a Separate one-way ANOVAs were performed on each root trait (*n* = 16 for each cropping system).

10.1128/mSystems.00651-21.1 TABLE S1 Effects of cropping systems on bulk soil physicochemical properties. Download Table S1, PDF file, 0.08 MB. Copyright © 2021 Bay et al. 2021 Bay et al. https://creativecommons.org/licenses/by/4.0/ This content is distributed under the terms of the Creative Commons Attribution 4.0 International license.

### Microbial community structure.

**FIG 1:**
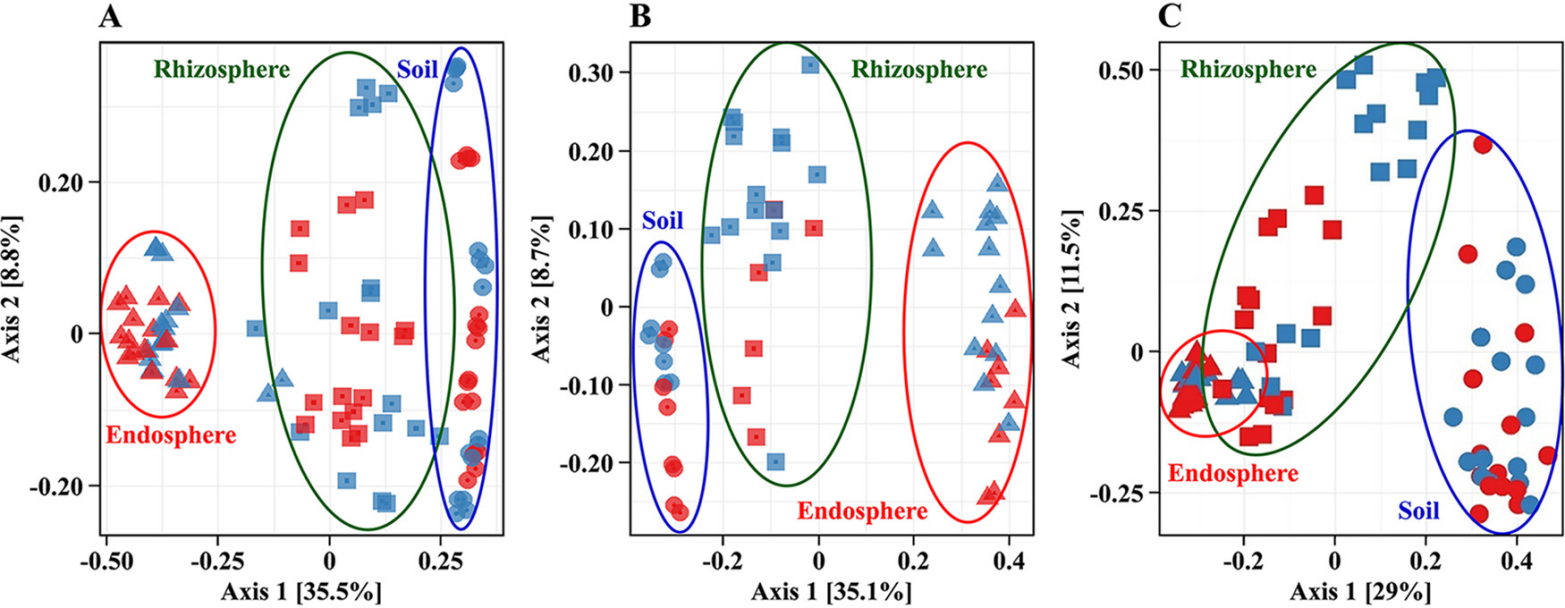
Bray-Curtis dissimilarity distance principal-coordinate analysis (PCoA) plots of total bacterial resident (DNA-based) (A), metabolically active (RNA-based) (B), and total fungal (C) resident community profiles of maize grown in bulk soil from conventional (red) and diversified (blue) cropping systems. Symbols: ●, bulk soil; ■, rhizosphere; ▲, endosphere.

Cropping system and root proximity (i.e., whether samples originated from the bulk soil, rhizosphere, or endosphere) influenced prokaryotic and fungal community composition, with some significant interactions (Table 2). Prokaryotic and fungal communities separate clearly by proximity to the roots (horizontal spread), and less clearly by cropping system (vertical spread) (Fig. 1). The effect of the cropping system on prokaryotic communities was more clearly resolved at the RNA level than the DNA level, where soil, rhizosphere, and endosphere samples generally clustered separately by cropping system; similar trends were also observed in the separation by proximity to the roots (Fig. 1A and B). Likewise, the fungal community was more clearly resolved by root proximity, and to a lesser degree by cropping system (Fig. 1C).

**TABLE 2:** Permutational multivariate analysis of variance of microbial β-diversity

Factor	Prokaryotic		Fungal	
	*R* ^2^	*P* value	*R* ^2^	*P* value
Cropping system	0.043	<0.001	0.074	<0.001
Root proximity^*a*^	0.327	<0.001	0.326	<0.001
Cropping system × root proximity^*a*^	0.024	<0.001	0.054	<0.001
DNA vs RNA	0.070	<0.001		
Cropping system × DNA vs RNA	0.006	0.070		
Root proximity^*a*^ × DNA vs RNA	0.035	<0.001		
Cropping system × DNA vs RNA × root proximity^*a*^	0.007	0.388		

a Root proximity tests whether there are differences between bulk soil, rhizosphere, and endosphere.

### Microbial diversity.

**FIG 2:**
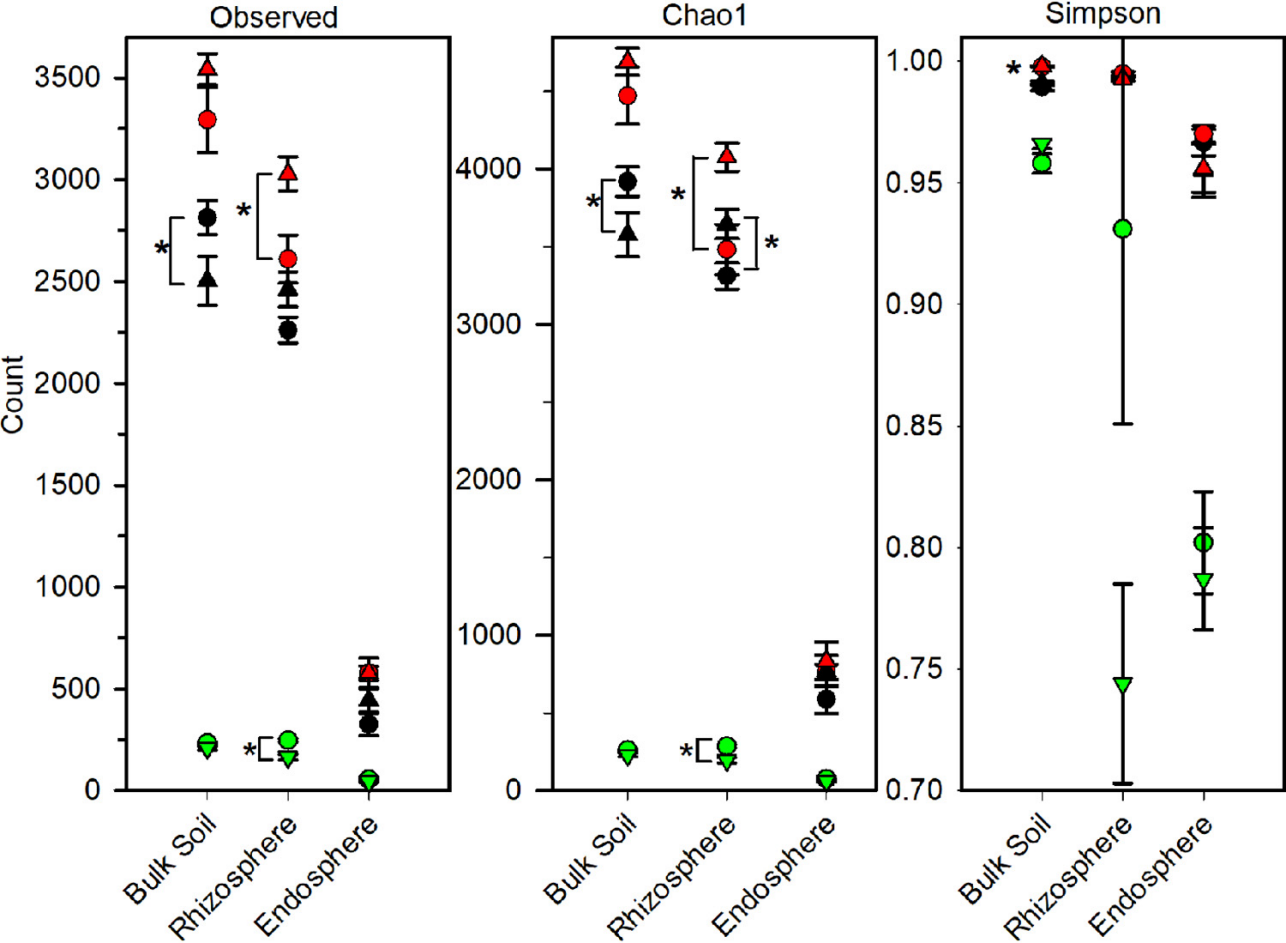
Richness (observed and Chao1) and Simpson’s evenness diversity indices in bulk soil, rhizosphere, and endosphere. The prokaryote metabolically active community (red), prokaryote total resident community (black), and fungi total resident community (green) are shown. Asterisks indicate statistically significant differences between cropping systems based on a Fisher’s LSD (*P* < 0.05) *post hoc* test. While not visible, evenness in the soil prokaryotic community was greater in the diversified than the conventional cropping system. ▴, diversified; ●, conventional.

The diversity of the microbial communities differed by proximity to the roots and by cropping system (Fig. 2 and Table S2). Root proximity tended to decrease prokaryotic species richness and to increase evenness. On the other hand, interpretation of whether diversified systems altered these two diversity indices was influenced by whether relying on DNA- or RNA-based analyses (Fig. 2 and Table 3), with RNA-based profiles indicating greater prokaryotic richness in diversified systems. Moreover, DNA-based prokaryotic species richness was also greater in the diversified system, with the exception that the bulk soil had higher richness in the DNA-based analyses. In contrast, the overall trend was the opposite for fungi, and the richness in the rhizosphere was significantly greater in the conventional system (Fig. 2). Evenness in the bulk soil differed between cropping systems for the RNA- but not DNA-based analyses, with a slight trend for increased evenness in the rhizosphere and endosphere of plants grown in the diversified cropping system. While the endosphere had a lower richness overall (both for prokaryotes and fungi), its community was more even, with little difference between DNA- and RNA-based analyses for either cropping system.

**TABLE 3:** Contrasts comparing prokaryotic DNA- and RNA-based α-diversity indices^*a*^

Root proximity	*P* value					
	Observed		Chao1		Simpson	
	Conv	Div	Conv	Div	Conv	Div
Bulk soil	0.008	<0.001	0.008	<0.001	<0.001	<0.001
Rhizosphere	0.050	<0.001	0.431	0.012	0.186	0.803
Endosphere	0.001	0.183	0.198	0.981	0.951	0.405

a Conv and Div refer to conventional and diversified cropping systems, respectively.

10.1128/mSystems.00651-21.2 TABLE S2 Analysis of variance of α-diversity indices. Download Table S2, PDF file, 0.1 MB. Copyright © 2021 Bay et al. 2021 Bay et al. https://creativecommons.org/licenses/by/4.0/ This content is distributed under the terms of the Creative Commons Attribution 4.0 International license.

### Differential abundance of prokaryotic communities.

**FIG 3:**
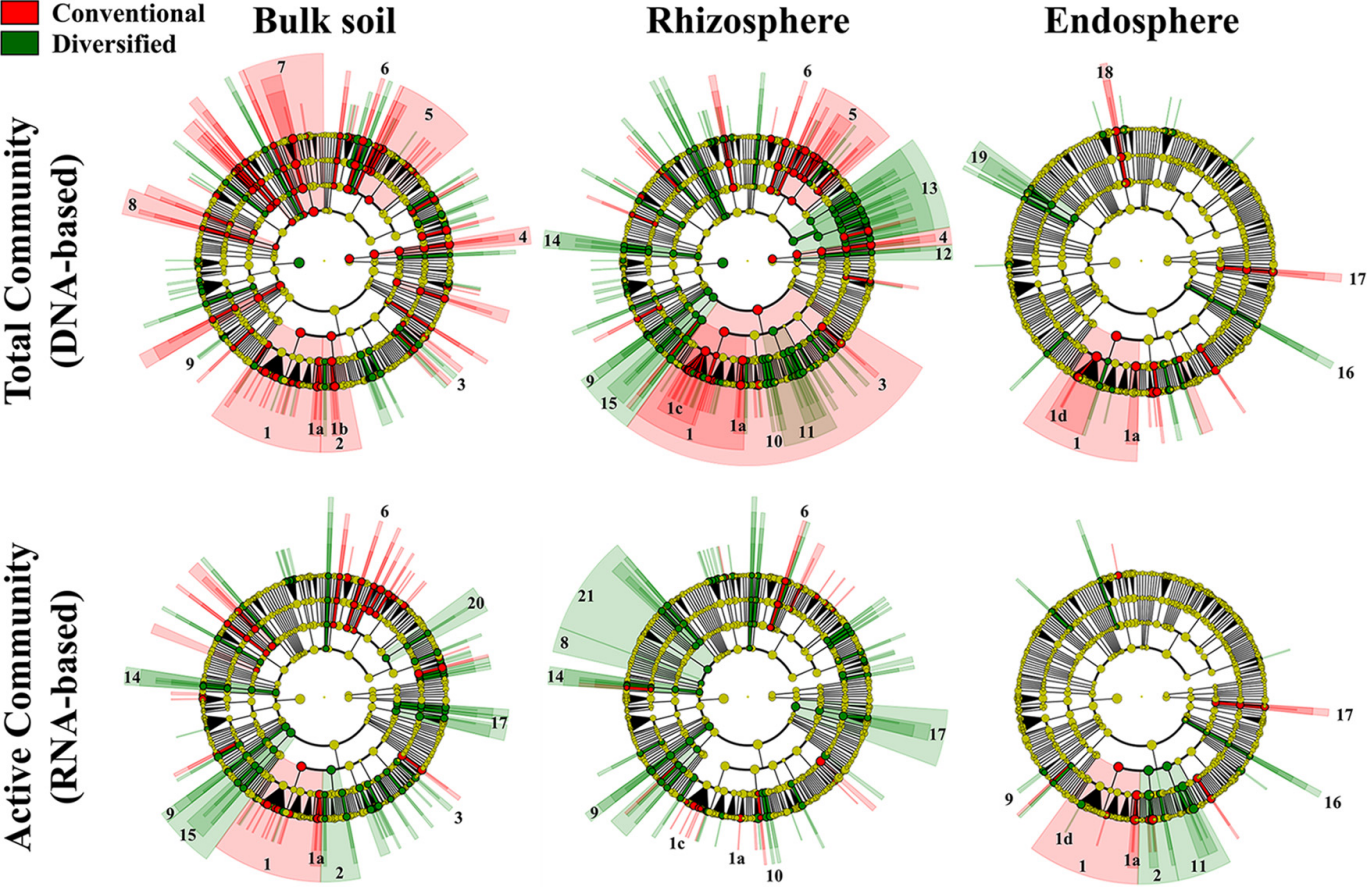
LDA effect size cladograms comparing the total resident (DNA-based) and metabolically active (RNA-based) prokaryotic community profiles. Separate analyses were performed on bulk soil, maize rhizosphere, and maize endosphere. Circles represent phylogenetic levels from kingdom to family from the center outwards. The colored nodes and shading denote significant differences in relative abundance between specific taxa in the conventional (red) or diversified (green) system; yellow node, no significant difference. 1, *Alphaproteobacteria* (1a, *Sphingomonadaceae* and *Erythrobacteraceae*; 1b, *Burkholderiales*; 1c, *Rhizobiaceae*; 1d, *Bradyrhizobiaceae*); 2, *Betaproteobacteria*; 3, *Pseudomonadales*; 4, *Thaumarchaeota*; 5, *Actinobacteria*; 6, *Rubrobacteria*; 7, *Bacteroidetes*; 8, *Cyanobacteria*; 9, *Nitrospiraceae*; 10, *Nitrosomonadaceae*; 11, Deltaproteobacteria; 12, *Euryarchaeota*; 13, *Acidobacteria*; 14, *Elusimicrobia*; 15, *Planctomycetes*; 16, *Spirochaetae*; 17, *Verrucomicrobia*; 18, *Flavobacteria*; 19, *Ktedonobacteria*; 20, *Holophagae*; 21, *Chloroflexi*.

**FIG 4:**
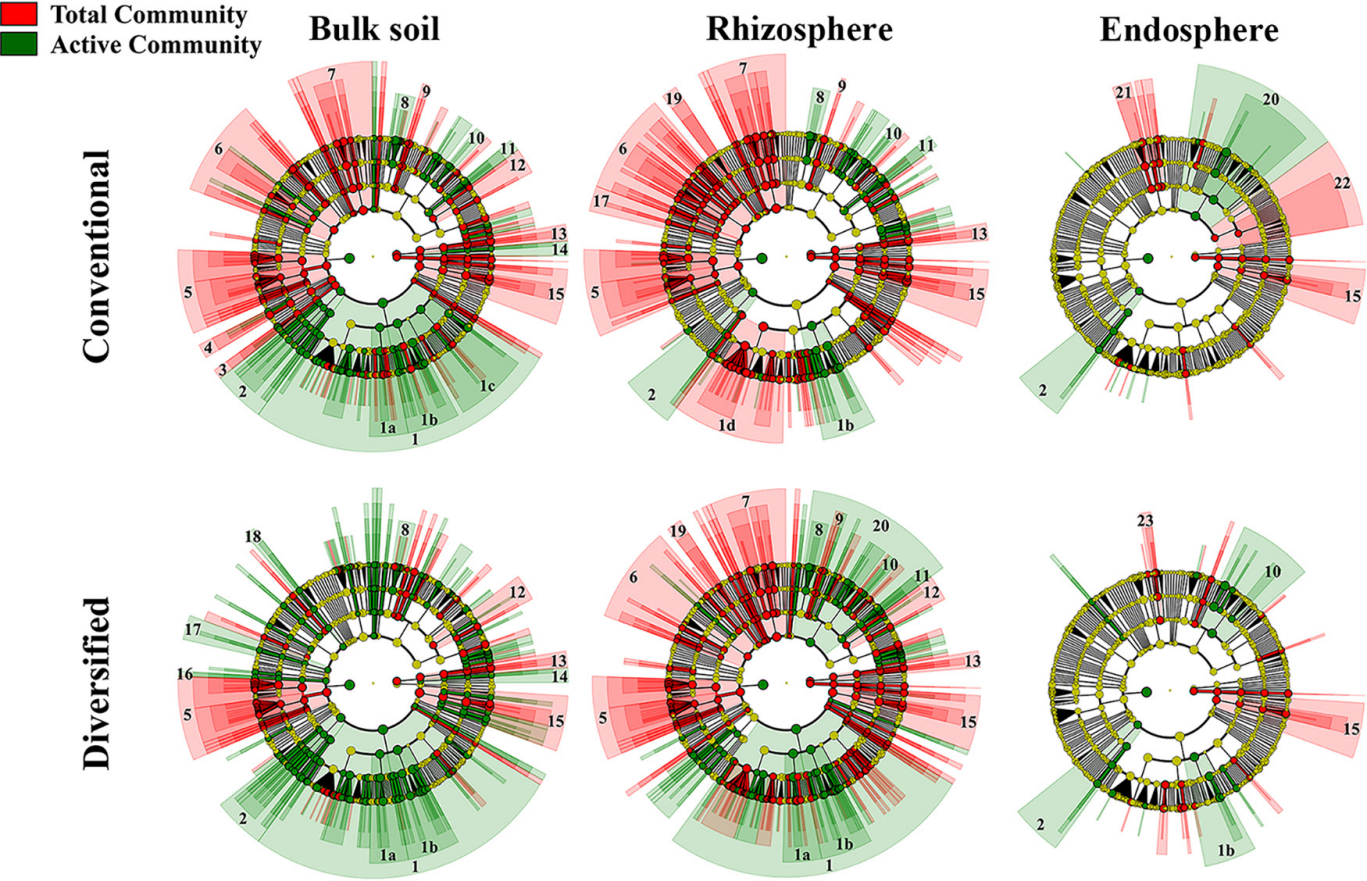
LDA effect size cladograms comparing the conventional and diversified cropping system prokaryotic community profiles. Separate analyses were performed on soil, maize rhizosphere, and maize endosphere. Colored nodes and shading denote significant differences in relative abundance between specific taxons in the total (red) or metabolically active (green) community; yellow node, no significant difference. 1, *Proteobacteria* (1a, *Betaproteobacteria*; 1b, Deltaproteobacteria; 1c, *Gammaproteobacteria*; 1d, *Alphaproteobacteria*); 2, *Planctomycetes*; 3, *Nitrospirae*; 4, *Gemmatimonadetes*; 5, *Firmicutes*; 6, *Chloroflexi*; 7, *Bacteroidetes*; 8, *Solirubrobacterales*; 9, *Rubrobacteria*; 10, *Frankiales*; 11, *Acidimicrobiia*; 12, *Holophagae*; 13, *Thaumarchaeota*; 14, *Euryarchaeota*; 15, *Verrucomicrobia*; 16, *Fibrobacteres*; 17, *Cyanobacteria*; 18, *Chlorobi*; 19, *Chlamydiae*; 20, *Actinobacteria*; 21, *Sphingobacteriia*; 22, *Acidobacteria*; 23, *Cytophagia*.

**FIG 5:**
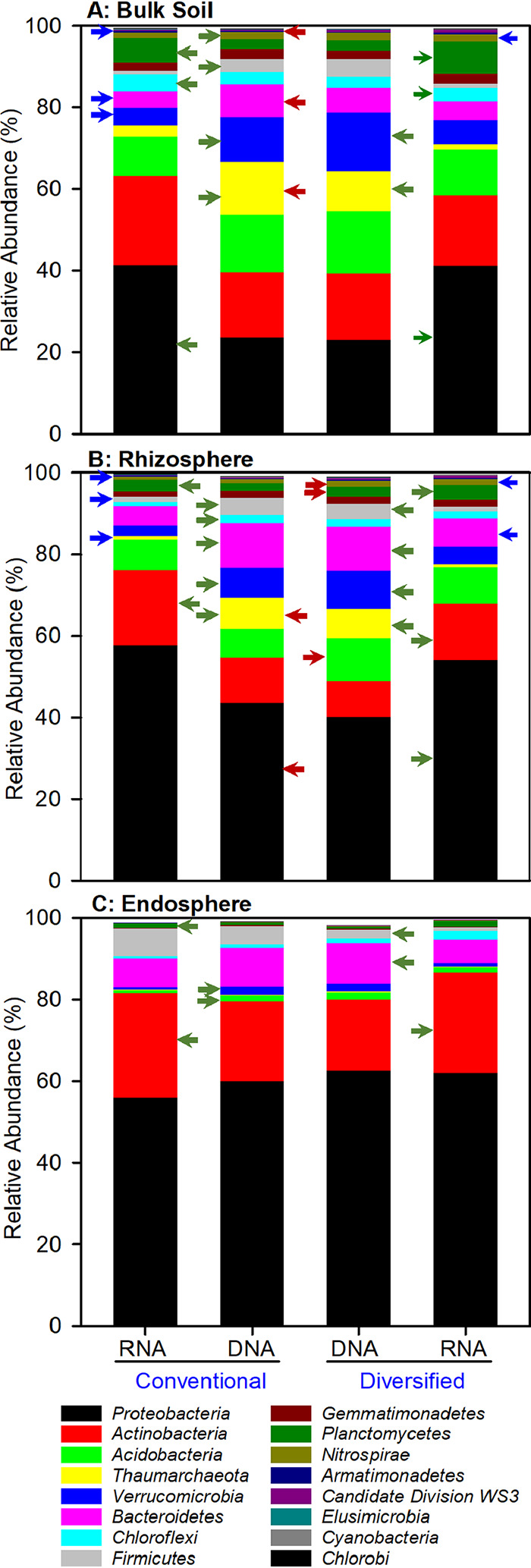
Phylum-level relative abundance of DNA- and RNA-based prokaryotic community profiles in conventional and diversified cropping systems. Red arrows identify statistically different (LDA, *P* < 0.05) DNA-based relative abundance between cropping systems. Green arrows indicate statistically different (LDA, *P* < 0.05) relative abundances between DNA- and RNA-based profiles within a cropping system. Blue arrows indicate statistically different (LDA, *P* < 0.05) RNA-based relative abundances between cropping systems. Low-abundance phyla are not included.

We performed linear discriminant effect size (LEfSe) analyses (35) at various taxonomic ranks to reveal how cropping system influenced prokaryotic communities (Fig. 3 and 4) and whether the total resident (DNA-based) and potentially metabolically active (RNA-based) community profiles differed (Fig. 5). Family level cladograms displaying significant enrichment of taxa (linear discriminant analysis [LDA], *P* < 0.05) are strikingly different, regardless of cropping system and DNA-/RNA-based profiles, most notably in the rhizosphere (Fig. 3 and 4). Cropping systems influenced the correspondence between DNA- and RNA-based profiles, particularly the bulk soil and rhizosphere communities (Fig. 5). While plant selection for taxa was generally similar, unique taxa were preferentially enriched in each cropping system.

### Bulk soil.

**FIG 6:**
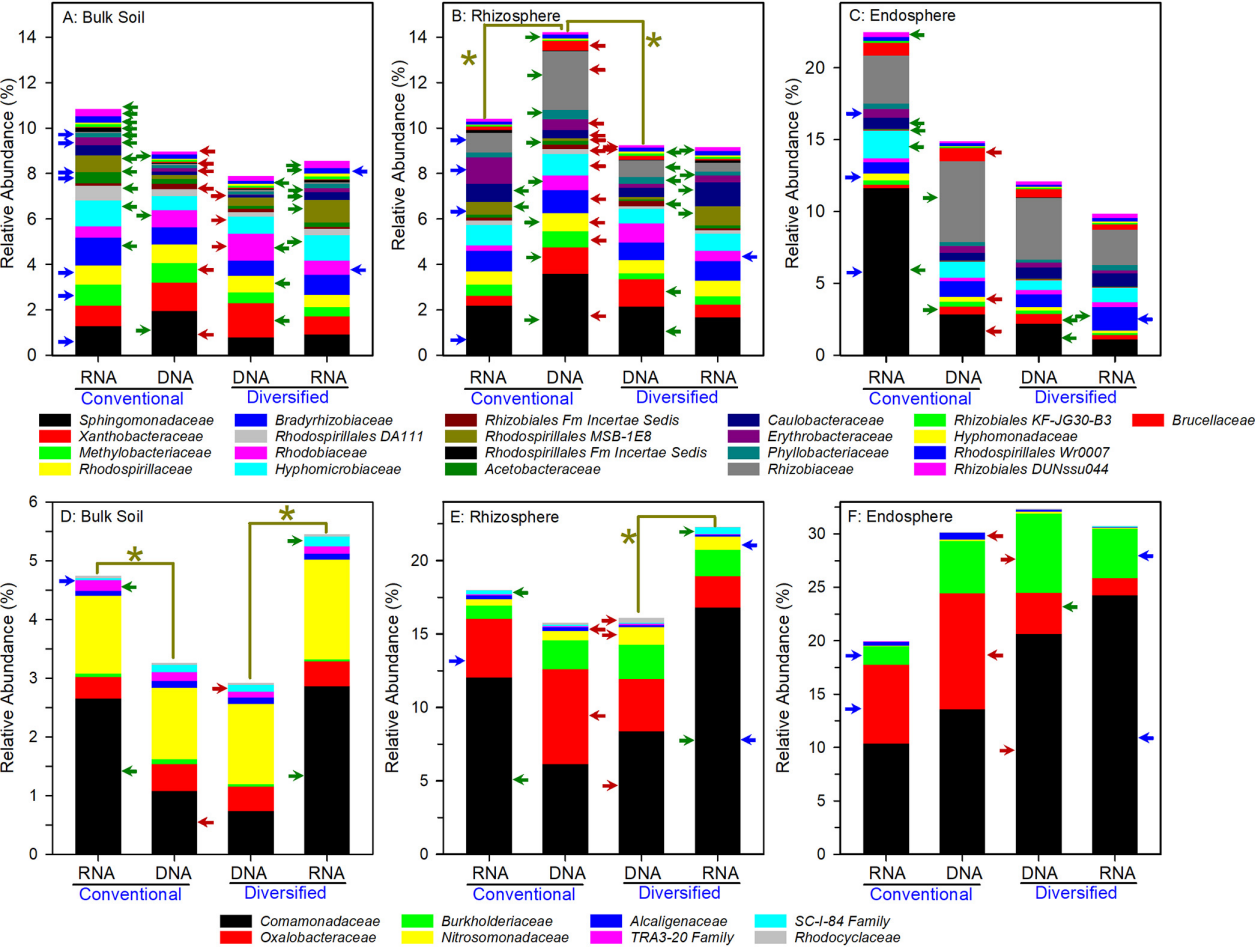
DNA- and RNA-based family level relative abundance of *Alphaproteobacteria* (A to C) and *Betaproteobacteria* (D to F) in conventional and diversified cropping systems. Red, green, and blue arrows are as defined in the legend to Fig. 5. An asterisk over two adjacent bars indicates statistically significant differences between RNA- and DNA-based analyses. Low-abundance families are not visible.

The bulk soil of each cropping system harbored distinct DNA- and RNA-based community profiles (Fig. 3, 4, and 5). At the DNA level, there were more differentially abundant phyla and families in the conventional soil of which 59% (30 out of 51 families) were *Proteobacteria* and *Actinobacteria* families (Table 4 and Fig. 6A and D; see also Fig. S1A and S1D and Fig. S2A and S2D in the supplemental material). Twelve out of the 19 differentially abundant proteobacterial families were *Alphaproteobacteria*, primarily of the order *Rhizobiales*. In contrast, in the diversified system, there were fewer differentially abundant families, representing fewer phyla; *Proteobacteria* and *Actinobacteria* represented ∼40% of the differentially abundant taxa (Table 4), whereas no proteobacterial class dominated (Fig. 3, Fig. 6A and D, Fig. S1A and S1D, and Fig. S2A and S2D). The *Actinobacteria* and *Acidobacteria* contributed uniquely to differences between systems, with 8 differentially abundant *Acidobacteria* families in the diversified system compared to 11 actinobacterial families in the conventional system (Fig. S2A and S2D); many were of low abundance. While there were only a few differences in *Planctomycetes* and *Verrucomicrobia* (Fig. S3A and S3D), the conventional system had more differentially abundant *Bacteroidetes* (Fig. S4A), whereas the diversified system had more differentially abundant *Firmicutes* families (Fig. S4D).

**TABLE 4:** Number of differentially abundant taxa at various taxonomic ranks^*a*^

Community^*b*^	Cropping system	Bulk soil		Rhizosphere		Endosphere	
		Phylum/class^*c*^	Family^*d*^	Phylum/class	Family	Phylum/class	Family
DNA	Conventional	6	51 (13)	2	41 (9)	0	8 (3)
	Diversified	1	43 (10)	7	72 (15)	1	17 (7)

RNA	Conventional	0	30 (8)	0	23 (5)	0	9 (4)
	Diversified	3	41 (12)	6	46 (13)	1	14 (7)

ITS	Conventional	4	16 (4)	5	22 (4)	1	2 (1)
	Diversified	3	12 (4)	3	7 (3)	1	2 (1)

a Based on individual LEfSe analyses comparing the conventional and diversified cropping systems.

b DNA, RNA, and ITS refer to the total resident prokaryotic, potentially metabolically active prokaryotic, and total resident fungal communities, respectively.

c Differentially abundant bacterial phyla or fungal classes.

d Values in parentheses are the number of phyla represented by the differentially abundant families.

10.1128/mSystems.00651-21.6 FIG S1 DNA- and RNA-based family level relative abundance of Deltaproteobacteria (A to C) and *Gammaproteobacteria* (D to F) in conventional and diversified cropping systems. Red arrows indicate statistically different (LDA, *P* < 0.05) DNA-based relative abundance between cropping systems. Green arrows indicate statistically different (LDA, *P* < 0.05) relative abundance between DNA- and RNA-based profiles within a cropping system. Blue arrows indicate statistically different RNA-based relative abundance between cropping systems. An asterisk over two adjacent bars indicates statistically significant different total relative abundance between the RNA- and DNA-based analyses. Low-abundance (<0.02%) *Delta*- and *Gammaproteobacteria* families are not visible. Download FIG S1, JPG file, 1.1 MB. Copyright © 2021 Bay et al. 2021 Bay et al. https://creativecommons.org/licenses/by/4.0/ This content is distributed under the terms of the Creative Commons Attribution 4.0 International license.

10.1128/mSystems.00651-21.7 FIG S2 DNA- and RNA-based family level relative abundance of *Actinobacteria* (A to C) and *Acidobacteria* (D to F) in conventional and diversified cropping systems. Red arrows indicate statistically different (LDA, *P* < 0.05) DNA-based relative abundance between cropping systems. Green arrows indicate statistically different (LDA, *P* < 0.05) relative abundance between DNA- and RNA-based profiles within a cropping system. Blue arrows indicate statistically different RNA-based relative abundance between cropping systems. An asterisk over two adjacent bars indicates statistically significant different total relative abundance between the RNA- and DNA-based analyses. Low-abundance *Actinobacteria* (<0.08%) and *Acidobacteria* (<0.05% for soil and rhizosphere; <0.01% for endosphere) families are not visible. Download FIG S2, JPG file, 2.0 MB. Copyright © 2021 Bay et al. 2021 Bay et al. https://creativecommons.org/licenses/by/4.0/ This content is distributed under the terms of the Creative Commons Attribution 4.0 International license.

10.1128/mSystems.00651-21.8 FIG S3 DNA- and RNA-based family level relative abundance of *Planctomycetes* (A to C) and *Verrucomicrobia* (D to F) in conventional and diversified cropping systems. Red arrows indicate statistically different (LDA, *P* < 0.05) DNA-based relative abundance between cropping systems. Green arrows indicate statistically different (LDA, *P* < 0.05) relative abundance between DNA- and RNA-based profiles within a cropping system. Blue arrows indicate statistically different RNA-based relative abundance between cropping systems. An asterisk over two adjacent bars indicates statistically significant different total relative abundance between the RNA- and DNA-based analyses. Low-abundance *Planctomycetes* (<0.05% for soil and rhizosphere; <0.005% endosphere) and *Verrucomicrobia* (<0.05% for soil and rhizosphere; <0.02% for endosphere) families are not visible. Download FIG S3, JPG file, 1.4 MB. Copyright © 2021 Bay et al. 2021 Bay et al. https://creativecommons.org/licenses/by/4.0/ This content is distributed under the terms of the Creative Commons Attribution 4.0 International license.

10.1128/mSystems.00651-21.9 FIG S4 DNA- and RNA-based family level relative abundance of *Bacteroidetes* (A to C) and *Firmicutes* (D to F) in conventional and diversified cropping systems. Red arrows indicate statistically different (LDA, *P* < 0.05) DNA-based relative abundance between cropping systems. Green arrows indicate statistically different (LDA, *P* < 0.05) relative abundance between DNA- and RNA-based profiles within a cropping system. Blue arrows indicate statistically different RNA-based relative abundance between cropping systems. An asterisk over two adjacent bars indicates statistically significant different total relative abundance between the RNA- and DNA-based analyses. Low-abundance *Bacteroidetes* (<0.02%) and *Firmicutes* (<0.01%) families are not visible. Download FIG S4, JPG file, 1.4 MB. Copyright © 2021 Bay et al. 2021 Bay et al. https://creativecommons.org/licenses/by/4.0/ This content is distributed under the terms of the Creative Commons Attribution 4.0 International license.

The RNA-based profiles revealed 33% more differentially abundant families representing 50% more phyla in the diversified bulk soil than in the conventional bulk soil (Table 4). The *Proteobacteria*, *Actinobacteria*, and *Acidobacteria* represent 51% of the differentially abundant taxa, the remainder of which are from nine other phyla (Table 4, Fig. 6A and D, Fig. S1A and S1D, and Fig. S2A and S2D). In the conventional bulk soil, these same three phyla comprise 73% of the differentially abundant families. Most of the differentially abundant *Proteobacteria* were *Alphaproteobacteria* (primarily *Rhizobiales* and *Rhodospirillales*), similar to what was observed with the DNA-based profiles (Fig. 6A). The increase in potentially metabolically active *Betaproteobacteria* was due primarily to the *Comamonadaceae* (Fig. 6D). There were few differences in the abundance of Deltaproteobacteria and *Gammaproteobacteria* (Fig. S1A and S1D), although Deltaproteobacteria were more abundant in the RNA-based profiles. The *Actinobacteria* were more potentially metabolically active in the conventional soils than in the diversified soils (Fig. S2A). As has been observed in various cropping systems (36, 37) and here in bulk soil and rhizosphere, the *Planctomycetes* were more abundant in the RNA- than DNA-based profiles (Fig. S3A and S3B), including the soil anammox clade *OM190* (36). Few differences were found in *Verrucomicrobia*, *Bacteroidetes*, and *Firmicutes* (Fig. S3D and Fig. S4A and S4D).

### Rhizosphere.

The rhizosphere of each cropping system harbored distinct DNA- and RNA-based community profiles (Fig. 3, 4, and 5), and the differences were particularly pronounced compared to bulk soil (Table 4). At the DNA level, *Actinobacteria*, *Acidobacteria*, and *Proteobacteria* comprised 50% and 76% of the differentially abundant families in the diversified and conventional rhizospheres, respectively. A key commonality with bulk soil was the preponderance of differentially abundant alphaproteobacterial families in the conventional (15 out of 18 *Proteobacteria*) compared to the diversified (8 out of 23 *Proteobacteria*) rhizospheres (Fig. 6B and D and Fig. S1B and S1D), including a >3-fold increase in *Rhizobiaceae* (Fig. 6B). Relative to bulk soil, the greatest enrichment in the rhizosphere were the *Betaproteobacteria*, and unlike in soil, several *Betaproteobacteria* families were more abundant in the RNA-based profiles in the diversified system (Fig. 6E). The *Comamonadaceae* and *Oxalobacteraceae* of the *Burkholderiales* were enriched in the diversified and conventional rhizospheres, respectively, but not the *Burkholderiaceae* (Fig. 6E). As in bulk soil, the Deltaproteobacteria were highly metabolically active in the rhizosphere, with some families exhibiting 2- to 5-fold differences between RNA- and DNA-based relative abundance (Fig. S1B), including the nitrite-oxidizing *Nitrospinaceae*. There were more *Pseudomonadaceae* in the conventional rhizosphere; in bulk soil, *Pseudomonadaceae* were more abundant in the diversified system (Fig. S1E).

Compared to bulk soil, there was an ∼33% decrease in *Actinobacteria* in the rhizosphere but unlike bulk soil, there were significant increases in RNA relative abundance, particularly in the conventional system (Fig. S2B). Similarly, both cropping systems were enriched in metabolically active *Actinobacteria* families. Furthermore, in both DNA- and RNA-based profiles, there were more differentially abundant *Acidobacteria* in the diversified system, many of which were in low abundance (Fig. S2E). Relative to bulk soil, there was a modest overall decrease in the abundance of *Planctomycetes* and *Verrucomicrobia* in the rhizosphere, and a modest overall increase in *Bacteroidetes* and *Firmicutes*, with low-abundance taxa distinguishing the two systems for *Bacteroidetes* (Fig. S3B and S3E and Fig. S4B and S4E).

### Endosphere.

DNA- and RNA-based community profiles in the endosphere were more congruent than in the rhizosphere or bulk soil (Fig. 3, 4, and 5). This is likely a consequence of plant selection for bacteria capable of surviving intercellular spaces and lack of influence of relic DNA on community profiles. Yet, despite these similarities, there were several notable differences, with 28 families (9 phyla) being more abundant in the diversified, compared to 10 families (3 phyla) in the conventional system (Table 4), although many were low-abundance taxa (<0.1%).

Increased representation of *Proteobacteria* in the endosphere, relative to the rhizosphere, was cropping system specific (Fig. 6C and F and Fig. S1C and S1F). The *Rhizobiaceae* was particularly enriched in the conventional endosphere, similar to what was observed in the rhizosphere (Fig. 6C). Additionally, the *Comamonadaceae* and *Oxalobacteraceae* were enriched in the diversified and conventional endospheres, respectively (Fig. 6F). The *Xanthomonadaceae* were enriched in the endosphere relative to the rhizosphere (Fig. S1C and S1F). While the RNA-based relative abundance of *Proteobacteria* did not differ between the two cropping systems (Fig. 5C), there was a dramatic shift in community composition, with more *Beta*- and Deltaproteobacteria in the diversified and more *Alphaproteobacteria* (particularly the *Sphingomonadaceae*) in the conventional system (Fig. 3, Fig. 6C and F, and Fig. S1C and S1F).

The endosphere was enriched in *Actinobacteria* (*Micromonosporaceae*, *Streptomycetaceae*, and *Pseudonocardiaceae*) compared to the rhizosphere and soil, comprising a substantial proportion of the potentially active community, particularly in the conventional endosphere (Fig. 5 and Fig. S2C). The effect of cropping system on the dynamic selection process for growth *in planta* is also reflected in the differential enrichment of *Verrucomicrobia* and *Bacteroidetes* families (Fig. S3F and S4C). The conventional endosphere, however, was depleted in *Acidobacteria* (Fig. S2F) and *Planctomycetes* (Fig. S3C). Finally, the endosphere was enriched in *Paenibacillaceae*, likely reflecting their enrichment in the rhizosphere (Fig. S4F).

### Differential abundance of fungal communities.

**FIG 7:**
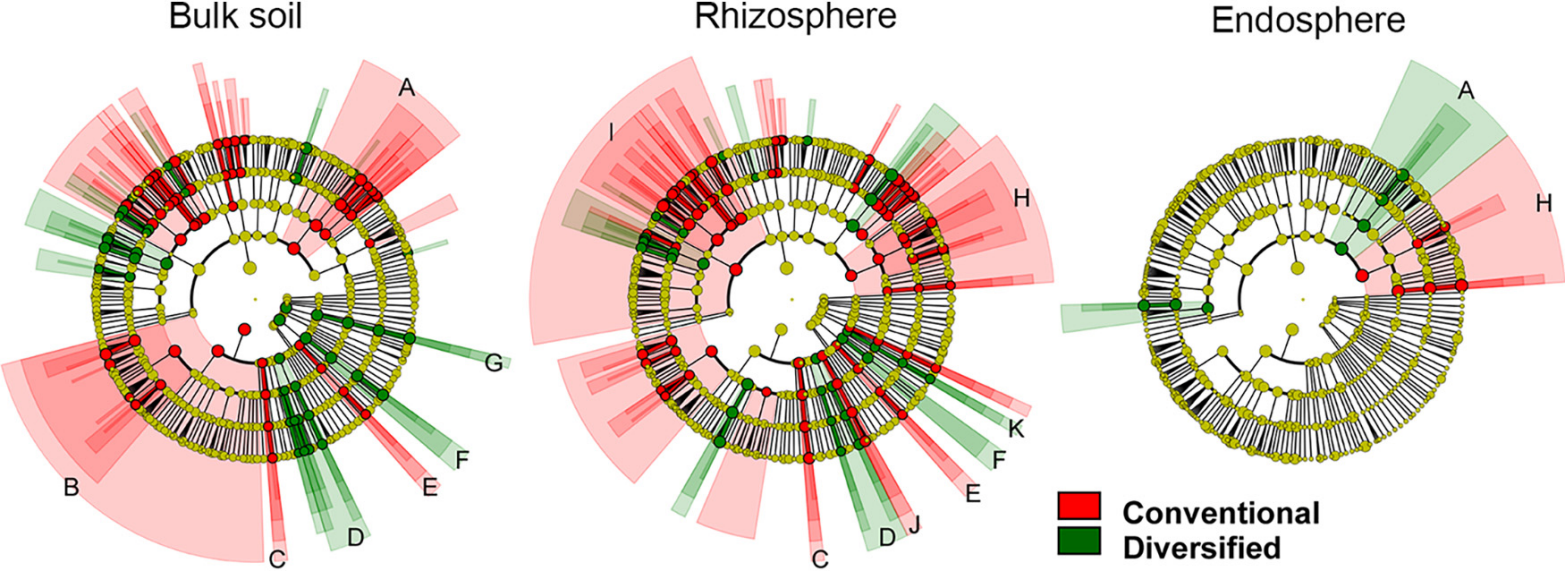
LDA effect size cladograms comparing the total resident (DNA-based) fungal community profiles, categorized by cropping system. Separate analyses were performed on bulk soil, maize rhizosphere, and maize endosphere. Circles represent phylogenetic levels from kingdom to family from the center outwards as described in the legend to Fig. 3. The colored nodes and shading denote significant difference in relative abundance between specific taxa in the conventional (red) or diversified (green) system; yellow node, no significant difference. A, *Eurotiomycetes*; B, *Agaricomycetes*; C, *Cystobasidiomycetes*; D, *Tremellomycetes*; E, *Lobulomycetes*; F, *Spizellomycetes*; G, unidentified class 14173; H, *Dothideomycetes*; I, *Sordariomycetes*; J, *Ustilaginomycetes*; K, *Glomeromycetes*.

Conventional and diversified soil were each uniquely enriched in various fungal families (Fig. 7 and Fig. S5). In the diversified bulk soil, there were fewer differentially abundant families (Table 4), of which 83% were *Ascomycota* (primarily *Sordariomycetes*) and *Basidomycota* (primarily *Tremellomycetes* and *Mortierellomycetes*). There was a pronounced cropping system effect in the rhizosphere (Fig. 7), leading to three times more differentially abundant fungal families in the conventional rhizosphere than in the diversified rhizosphere (Table 4), primarily *Ascomycota* and *Basidiomycota*. The conventional rhizosphere and endosphere were enriched in numerous phytopathogenic fungal families (*Botryosphaeriaceae*, *Glomerellaceae*, *Diaporthaceae*, *Pleosporaceae*, and *Ustilaginaceae*). Furthermore, the *Glomeromycetes* were significantly more abundant in the diversified rhizosphere than in the conventional rhizosphere. There were few differences between conventional and diversified endospheres; the conventional endosphere was enriched in plant pathogens, while the diversified endosphere was enriched in *Trichocomaceae* and *Microdochiaceae*, which are known saprophytes and plant pathogens, respectively.

10.1128/mSystems.00651-21.10 FIG S5 Class level relative abundance of fungal community profiles in conventional and diversified cropping systems. Arrows indicate statistically different (LDA, *P* < 0.05), DNA-based relative abundance of a class between the two cropping systems. Phyla below 1% were summed. Phyla are abbreviated as follows: Ascom, *Ascomycota*; Basid, *Basidiomycota*; Morti, *Mortierellomycota*; Chytr, *Chytridiomycota*; Glome, *Glomeromycota*. Download FIG S5, JPG file, 0.5 MB. Copyright © 2021 Bay et al. 2021 Bay et al. https://creativecommons.org/licenses/by/4.0/ This content is distributed under the terms of the Creative Commons Attribution 4.0 International license.

### Ammonia oxidizer community structure and nitrification.

**FIG 8:**
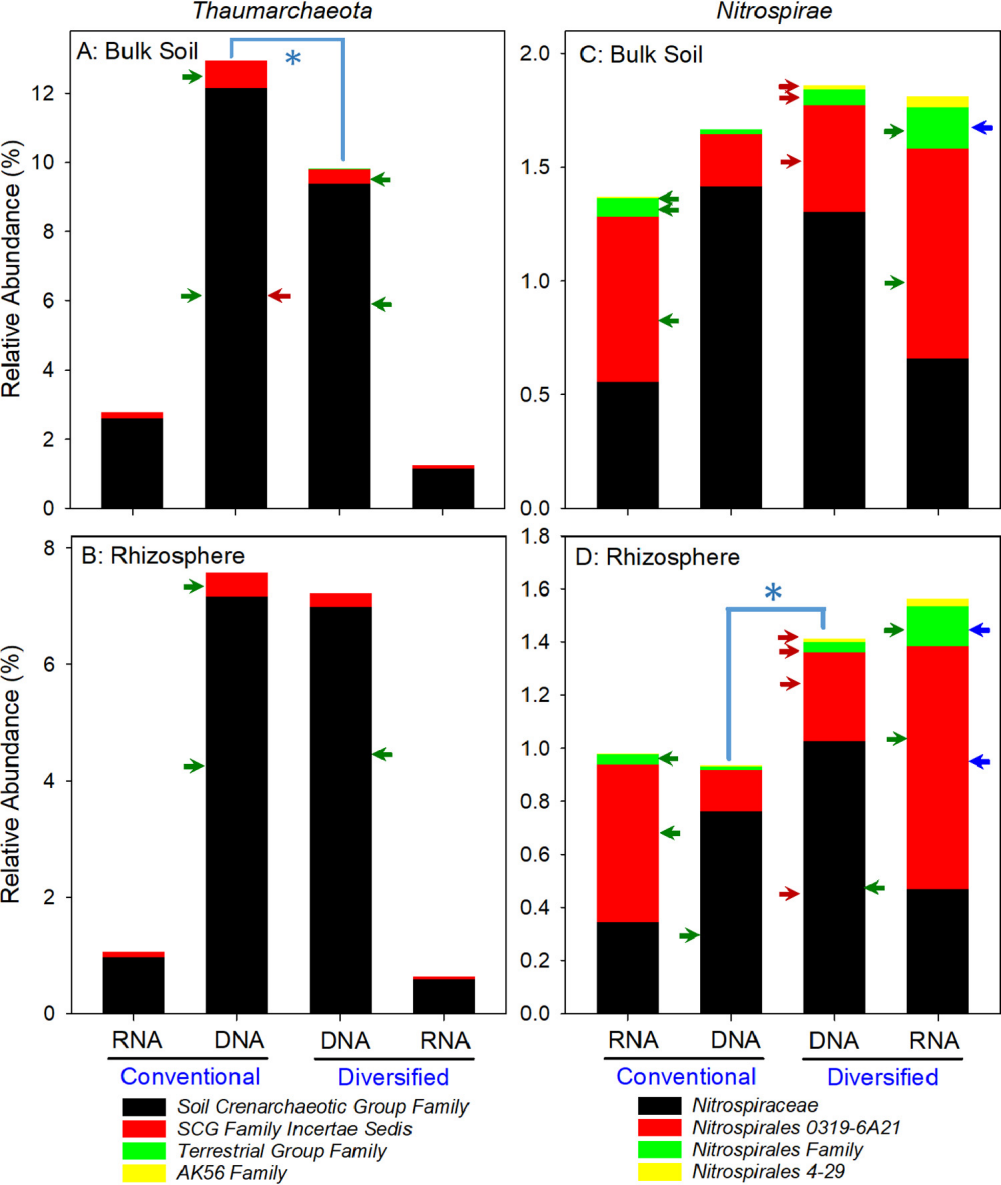
DNA- and RNA-based family level relative abundance of *Thaumarchaeota* and *Nitrospirae* in bulk soil and the rhizosphere of conventional and diversified cropping systems. (A to D) *Thaumarchaeota* (A and B); *Nitrospirae* (C and D). Red, green, and blue arrows are as defined in the legend to Fig. 5. An asterisk indicates statistically significant difference at the phylum level. Low-abundance families are not visible.

**FIG 9:**
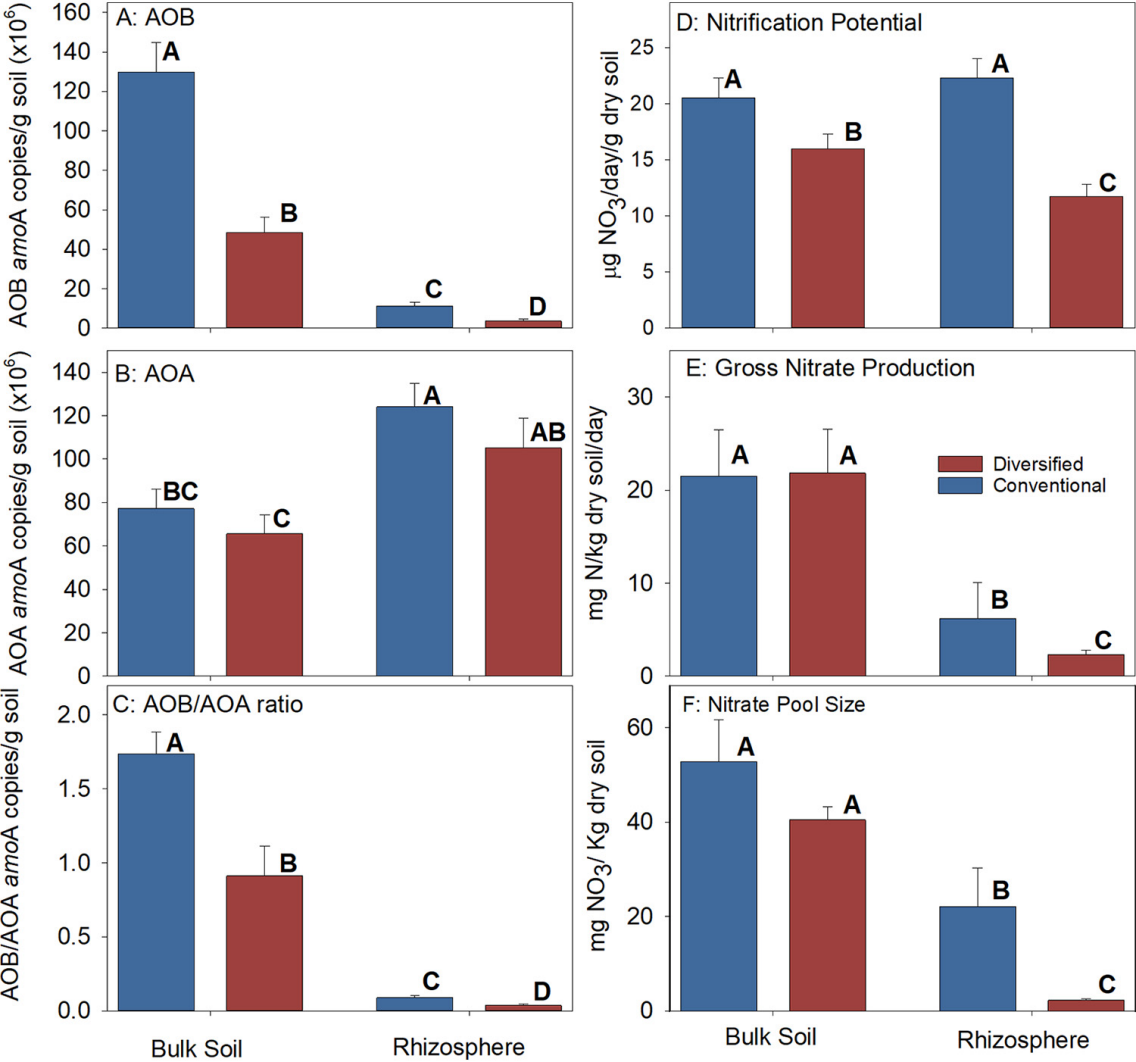
Root and cropping system influences on ammonia oxidizer abundance and nitrification. (A to F) AOB *amoA* gene abundance (A); AOA *amoA* gene abundance (B); AOB/AOA ratio (C); potential nitrification activity (D); gross nitrate production (^15^NO_3_ pool dilution) (E); nitrate pool sizes (F). Values are means plus standard errors of the means (SEM) (error bars). Separate two-way ANOVAs were performed on each treatment and are summarized in Table S3 in the supplemental material. Bars with different letters above the bars indicate significant differences at *P* < 0.05 based on Tukey’s HSD test.

For both the DNA- and RNA-based profiles, the *Nitrosomonadaceae* relative abundance was highest in bulk soil, and they were enriched in the diversified rhizosphere (Fig. 3 and 6D). There were more *Thaumarchaeota* in the conventional bulk soil, but there were no differences in the rhizosphere (Fig. 8A and B). While the *Nitrospiraceae* were enriched in the diversified rhizosphere, there was also enrichment of other *Nitrospirae* in both the diversified bulk soil and rhizosphere (Fig. 8C and D). We observed a cropping system but not rhizosphere effect in the enrichment of specific operational taxonomic units (OTUs). We also measured ammonia-oxidizing bacteria (AOB) and archaea (AOA) *amoA* gene abundance by quantitative PCR (qPCR). While it was not surprising to detect higher AOB *amoA* gene abundance in the conventional bulk soil than in the diversified bulk soil, given differences in inorganic N fertilizer inputs (4), it was interesting to observe a similar pattern in the rhizosphere (Fig. 9A). Our results also suggest that there is an enrichment of *Thaumarchaeota* in the rhizosphere, independent of cropping system effect (Fig. 9B and C), and that there may be more *Nitrosomonadaceae* in the conventional soil than expected based on relative abundance (Fig. 6D and 9A).

10.1128/mSystems.00651-21.3 TABLE S3 Analysis of variance of ammonia oxidizers and their potential metabolic activity. Download Table S3, PDF file, 0.09 MB. Copyright © 2021 Bay et al. 2021 Bay et al. https://creativecommons.org/licenses/by/4.0/ This content is distributed under the terms of the Creative Commons Attribution 4.0 International license.

Bulk soil nitrification potentials, gross nitrate production rates, and nitrate pool sizes did not differ substantially between cropping systems (Fig. 9D to F) at the V4/V5 developmental stage. In contrast, rhizosphere soils exhibited a cropping system effect, although the trend of lower gross nitrate production rates in the diversified rhizosphere was not statistically significant (Table S3). Despite no apparent correlation between *Thaumarchaeota*, *Nitrosomonadaceae*, or *Nitrospira* relative abundance and potential nitrification rates, patterns in nitrate pool sizes and gross nitrate production rates were more similar to the AOB/AOA *amoA* gene abundance ratio (Fig. 9C to F), indicating the relationship between these two groups of organisms could explain the lower levels of nitrate detected in subsurface water in the diversified system (23).

## DISCUSSION

We investigated the effects of conventional (2-year rotation, inorganic fertilization) and diversified (4-year rotation, manure amendments) cropping systems on the assembly of the prokaryotic and fungal root-associated communities at a maize developmental stage when N demand is poised to increase. The premise was that plant selection for a microbial community facilitating nutrient acquisition or retention would likely be in place before the onset of rapid nutrient uptake by the plant. We assessed whether cropping system influenced rhizosphere priming of the prokaryotic community by comparing the total resident (DNA-based) with the potentially metabolically active (RNA-based) community profiles, as well as its effects on gross rates and potential of nitrification. While our extraction protocols are considered nonquantitative, differences in taxon relative abundances do reflect differences in community composition. The root-soil compartments examined reflect the continuum from the soil to the plant, an important consideration when exploring the phytobiome (38). Our results show that due to the influence of agricultural management on the soil microbial community, the rhizosphere and endosphere microbial communities of diversified systems are distinct, as are the potential metabolic activities of rhizosphere communities.

DNA-based profiles reflect a range of metabolic states (dormant, growing) and relic (dead) while RNA analyses reflect the potentially metabolically active state (potential for protein synthesis) (33). The potential for protein synthesis likely reflects the ability of an organism to rapidly respond to changes in the environment (33, 39). Compositional changes in RNA profiles occur in response to nutrient inputs, including root exudates (40–42), and active communities have been shown to correlate better with CO_2_ production following a rain event (43) than total community composition. Prokaryotic species richness was higher in the RNA- than DNA-based analyses, suggesting that RNA-based profiling may provide greater differentiation of how environmental factors influence community composition. The greater congruence between the DNA- and RNA-based profiles within and on the root may reflect a reduction in the potential influence of relic DNA on community profiles (44, 45) or the greater stimulation of metabolic activities by nutrients provided by the root (34, 40). This is consistent with a prior report of greater correspondence between DNA- and RNA-based profiles in the rhizoplane of maize and other plant species (34). Convergence of community indices (i.e., relative abundance and species richness/evenness) in the endosphere likely reflects the strong selective environment of living within plants (46–48). Thus, management influences the soil microbial seed bank from which the plant enriches specific populations via rhizodeposits in the rhizosphere, providing a niche-specific community from which endophytes are selected. Collectively, diversified systems promote greater prokaryotic species richness in the maize root-associated microbiome, possibly due to the effects of different crop species, manure amendments, and/or reduced tillage on microbial community structure (34, 47, 49, 50). This is consistent with reports showing that long-term chemical-only fertilization induces diversity decline of soil bacteria (51). In contrast, species richness of fungal rhizosphere communities was greater in conventional systems, suggesting management can influence fungal and prokaryotic species diversity differently.

Reduced N availability in the diversified system likely influences maize root architecture and, in turn, the rhizosphere microbial community structure. Fine roots are known to drive microbial community dynamics, as well as nutrient cycling and water acquisition (52, 53). Enhanced root formation may increase rhizodeposits, and with a larger, more ramified root system exploring a greater soil volume, more C is likely being provided to a larger proportion of soil microbes in the diversified system. This could partially explain the greater prokaryotic richness as well as differences in potential metabolic activity in the diversified rhizosphere. Consistent with this premise is the greater relative abundance of AMF (*Glomeromycetes*) in the diversified rhizosphere, although not within the root where arbuscules form. Whether there is greater AMF biomass in the rhizosphere, where fungal hyphae acquire nutrients, needs to be determined. These observations suggest that plants in diversified systems increase soil resource acquisition not only via roots, but also possibly via AMF that could tighten N coupling between plants and microbial processes (53), perhaps contributing to yield benefits in these systems. Future work should explore this further by better assessing AMF abundance and composition with AMF-specific primer sets as well as by measuring AMF hyphal biomass in the rhizosphere soil compared to bulk soil (29).

There were more prokaryotic families, representing a greater number of phyla, that were more abundant in the diversified rhizosphere than in the conventional rhizosphere, which likely influences differences in the assembly of the endosphere community. Enrichment of *Acidobacteria*, *Bacteroidetes*, and *Verrucomicrobia* families in the diversified rhizosphere compared to the conventional rhizophere is consistent with previous reports describing maize rhizoplane communities at this same experimental site (27). These organisms are known for the breakdown of complex organic materials, such as plant polysaccharides (54–57). In contrast, *Proteobacteria* and *Actinobacteria* were selectively enriched in the conventional rhizosphere, which likely influenced the preferential enrichment of the *Proteobacteria* in the endosphere. This is consistent with the notion that the conventional rhizosphere community is more reliant on simple C substrates (56). Considered together, this suggests that conventional systems are less suited to complex C decomposition and, by extension, ammonia production, which is consistent with previous findings about the organic matter dynamics at this site (4, 24, 26, 58).

In response to rhizodeposits, microbial growth in the rhizosphere increases, and this growth may stimulate microbial demand for N, leading to direct competition with the plant for N. This microbial demand for N could be met with increased N mineralization (ammonium production), decreased nitrification, or both (59–61). Prior work at the site used to collect soils for this study concluded that N mineralization likely did not contribute to the yield benefit of diversified cropping systems (24, 58). Here we show that nitrification (potential activity, gross rates) is suppressed more in the diversified rhizosphere than in the conventional rhizosphere at the V4/V5 developmental stage. This is consistent with another report indicating that potential nitrification activity is lower in the maize rhizosphere at an early reproductive stage (62) and with lower nitrate levels detected in soil water in the diversified system at this study site (23). Lower nitrification in the rhizosphere reflects a complex shift in nitrifier community structure, with proportionally more AOA relative to AOB in the rhizosphere than bulk soil, in a cropping system-specific manner. The trend of lower nitrification in the diversified rhizosphere is likely due to a combination of a shift toward a nitrifying community that is possibly more adept at subsisting on lower levels of ammonium, smaller populations of microbes that perform nitrification, and/or lower metabolic activity of nitrifying bacteria. While our data support a more efficient coupling of N supply with plant and microbial demand in the diversified rhizosphere, more in-depth exploration into how management and the rhizosphere influence N cycling activities is essential for confirming or refuting our conclusions.

In summary, we show that in a diversified cropping system at a stage at which the plant is poised for rapid nutrient uptake, the plant selected a more diverse prokaryotic community, with altered N cycling populations and their metabolism that may contribute to yield benefits and reduced N losses. While there is convergence in community structure during plant selection for bacterial endophytes, that structure is influenced by the rhizosphere community structure. In the diversified system, maize increased soil resource acquisition (more ramified root system, increased AMF relative abundance), which will alter the microbial habitat (sites for colonization, rhizodeposition patterns). One possible consequence of the altered habitat is the greater competition for ammonium during decomposition processes which could decrease nitrification. It is tempting to conclude that one mechanism by which the diversified system at the Marsden experimental site is able to support high productivity with reduced inorganic fertilization is more efficiently coupled C and N cycles. Yet this alone may not explain the yield benefit entirely, since plant health may be better in the diversified system due to decreased fungal pathogen pressure, as well as selection of a plant-associated microbiome which stimulates plant growth directly.

## MATERIALS AND METHODS

The following is a summarized version of Materials and Methods used in this study; the detailed version is available as Text S1 in the supplemental material.

10.1128/mSystems.00651-21.5 TEXT S1 Detailed Materials and Methods. Download Text S1, PDF file, 0.2 MB. Copyright © 2021 Bay et al. 2021 Bay et al. https://creativecommons.org/licenses/by/4.0/ This content is distributed under the terms of the Creative Commons Attribution 4.0 International license.

### Experimental site description, soil collection, and soil analyses.

Soil was collected from Iowa State University’s Marsden Long-term Cropping System Experiment, USA, in June 2014. Soils and management practices at the site have previously been described (4). We sampled from a conventional system (2-year rotation of maize and soybean) and a diversified system (4-year rotation of maize, soybean, oat/alfalfa, and alfalfa). There were four replicated blocks for each management system.

Five samples were aseptically collected in each corn plot; samples from the same plot were pooled and sieved; packing of rhizotrons occurred within 48 h of soil collection. Soil physicochemical properties are described in Table S1 in the supplemental material.

### Rhizotrons, growth conditions, and sampling protocols.

For each cropping system, two rhizotrons were filled with soil from each block to the average bulk density of the upper soil layer at the sampling site. Two surface-sterilized, pregerminated maize seedlings were planted in each rhizotron. The growth conditions were monitored (65% water-holding capacity, 24°C, 16-h/8-h light/dark photoperiod) until the plants reached vegetative stage V4/V5.

Bulk soil samples were aseptically collected (∼1 g; >1 cm from a root), and the root system of a plant was then extracted. The rhizosphere was obtained by vortexing and sonicating the roots in phosphate buffer (27). After pooling and filtering the washates/sonicates, the filtrate was centrifuged. The pellet obtained was resuspended in sterile water, centrifuged, and decanted to obtain a rhizosphere sample. Prior to obtaining endosphere samples, soil-free roots were sonicated in sterile water and scanned for automatic root image analysis (63). They were then freeze-dried, weighed, and pulverized for endosphere sampling. All samples were kept at −80°C.

### DNA and RNA extractions.

DNA and RNA were extracted from all samples with commercially available kits. After DNase treating the RNA extracts, first- and second-strand cDNA synthesis was performed. The DNA concentrations were determined by spectrophotometry, while the quality of the cDNA was assessed with a 2100 Bioanalyzer. All samples were stored at −80°C.

### Amplicon sequencing and processing.

For the 16S rRNA V4 region, amplicon library preparation and sequencing using the 515/806R primers (64) were performed by Argonne National Laboratory, USA. For the fungal internal transcribed spacer (ITS) region, DNA was sequenced at the University of Minnesota Genome Center using the ITS1/ITS2 primers. Amplicons of 16S rRNA and ITS were paired end sequenced (250 bp) on Illumina MiSeq instruments in separate runs. Three replicates of bacterial or fungal mock community DNA (65) were included with the respective libraries.

Following the removal of alien sequences (e.g., adapters, mitochondria), quality filtering (Q25), sample sorting, OTU clustering, and the filtering out of chimeras/singletons (66), taxonomy was assigned to the 16S rRNA sequences (67). They were then aligned (68), and a phylogenetic tree was produced in QIIME. A total of 5,453,802 reads were generated, with a median sequence length of 253 bp.

Similarly, an OTU table was generated for fungal ITS sequences after removal of alien sequences, quality filtering (Q25), phiX DNA filtering, sequence dereplication, and generation of a parametric error model (69). Chimeras were then removed, and taxonomy was assigned (70).

### Data analysis.

Distance matrices of 16S rRNA OTUs were created in QIIME using weighted and unweighted UniFrac distances (71), and Bray-Curtis dissimilarity distances, also used to examine ITS OTUs. All analyses were performed on nonrarefied data (72). Further analyses were carried out using PHYLOSEQ (73) and LEfSe (35). Based on the analysis of the mock bacterial communities (Table S4), family level resolution was used for identifying differentially abundant taxa, and OTU level resolution was used to quantify the total number of differentially abundant taxa.

10.1128/mSystems.00651-21.4 TABLE S4 Mock community identification at the genus level. Download Table S4, PDF file, 0.1 MB. Copyright © 2021 Bay et al. 2021 Bay et al. https://creativecommons.org/licenses/by/4.0/ This content is distributed under the terms of the Creative Commons Attribution 4.0 International license.

### Assessment of ammonia oxidizer abundance.

Assessment of AOA and AOB abundances was performed by targeting the *amoA* gene of DNA samples by qPCR (based on SYBR green fluorescence); the primer sets for AOB and AOA were *amoA*-1F/2R (74) and Arch-*amoA*F/R (75), respectively.

### Nitrification potential and gross nitrate production.

Separate rhizotrons, with and without maize plants, were prepared for determining the nitrification potential and gross nitrate production rates. Nitrification potential was determined over a 24-h period as described previously (76). For determining gross NO_3_ production rates, a ^15^KNO_3_ solution was first homogenously distributed in the soil of each rhizotron, samples were then collected after 15-min, 3-h, and 24-h incubation and immediately subjected to a KCl extraction. ^15^N/^14^N ratio determination occurred at Utah State University, as described previously (60, 77). The amount of ^15^NO_3_ to apply was determined by measuring inorganic N pool sizes in five rhizotrons (with and without plants). Subsamples of bulk and rhizosphere soils were collected for measuring soil physicochemical properties.

### Statistical analyses.

Amplicon data analyzed with permutational multivariate analyses of variance (PERMANOVAs) using distance matrices (78), and elemental and root traits analyzed with analyses of variance (ANOVAs) (in R, on transformed data [79–81]). ANOVAs of qPCR data, nitrification potentials, nitrate pool sizes, and gross nitrate production rates performed using JMP13 (on transformed data; *post hoc* test, Tukey’s honestly significant difference test [HSD]). Richness indices tested by ANOVAs (*post hoc* test, Fisher’s least significant difference [LSD]), and Simpson index examined by Kruskal-Wallis tests, in JMP13.

### Data availability.

All sequences were deposited into the NCBI Sequence Read Archive (PRJNA686799 and PRJNA685216).
