# Supplementary material for: Agricultural Management Affects the Active Rhizosphere Bacterial Community Composition and Nitrification
Source: mSystems. 2021 Sep 28;6(5):e00651-21. doi: 10.1128/mSystems.00651-21 (PMC8547420; doi:10.1128/mSystems.00651-21)
Supplement: TABLE S1 [file msystems.00651-21-st001.pdf]

Table S1. Effect of cropping systems on bulk soil physicochemical properties.

Property	Conventional Mean ± SEM	Diversified Mean ± SEM	ANOVA p-value
pH	6.5 ± 0.1	6.5 ± 0.1	0.99
Total Carbon (%)	2.7 ± 0.2	2.7 ± 0.3	0.98
Total Nitrogen (%)	0.20 ± 0.02	0.17 ± 0.02	0.98
Organic Matter (%)	5.0 ± 0.4	5.0 ± 0.5	0.99
Phosphorous (mg kg^{-1})	78.2 ± 6.1	61.2 ± 8.7	0.19
Potassium (mg kg^{-1})	177 ± 13	134 ± 10	**0.03**
Copper (mg kg^{-1})	145 ± 6	130 ± 13	0.55
Iron (mg kg^{-1})	2.0 ± 0.3	2.5 ± 0.4	0.53
Ammonium (mg kg^{-1})	2.8 ± 0.5	2.0 ± 0.4	0.19
Nitrate (mg kg^{-1})	30.3 ± 6.2	3.1 ± 0.3	**<0.001**

Mean ± SEM; n = 5 (pH) and n = 8 (macro- and micronutrients). Significant differences calculated by ANOVA are in bold. Statistical analysis performed on Box-Cox-transformed data.
