# Supplementary material for: Agricultural Management Affects the Active Rhizosphere Bacterial Community Composition and Nitrification
Source: mSystems. 2021 Sep 28;6(5):e00651-21. doi: 10.1128/mSystems.00651-21 (PMC8547420; doi:10.1128/mSystems.00651-21)
Supplement: TABLE S2 [file msystems.00651-21-st002.pdf]

Table S2 Analysis of variance of α-diversity indices.

	p-values		
16S rRNA	Observed	Chao1	Simpson
Cropping System	0.0627	0.0179	0.6057
DNA vs RNA	<0.001	<0.001	0.0288
Root proximity	<0.001	<0.001	<0.001
ITS			
Cropping System	<0.001	<0.001	0.094
Root proximity	<0.001	<0.001	<0.001

Separate ANOVAs were performed for Observed and Chao1 species richness α-diversity indices. A Kruskal-Wallis test was performed on the Simpson evenness index.
