# Supplementary figures and images for: Agricultural Management Affects the Active Rhizosphere Bacterial Community Composition and Nitrification
Source: mSystems. 2021 Sep 28;6(5):e00651-21. doi: 10.1128/mSystems.00651-21 (PMC8547420; doi:10.1128/mSystems.00651-21)

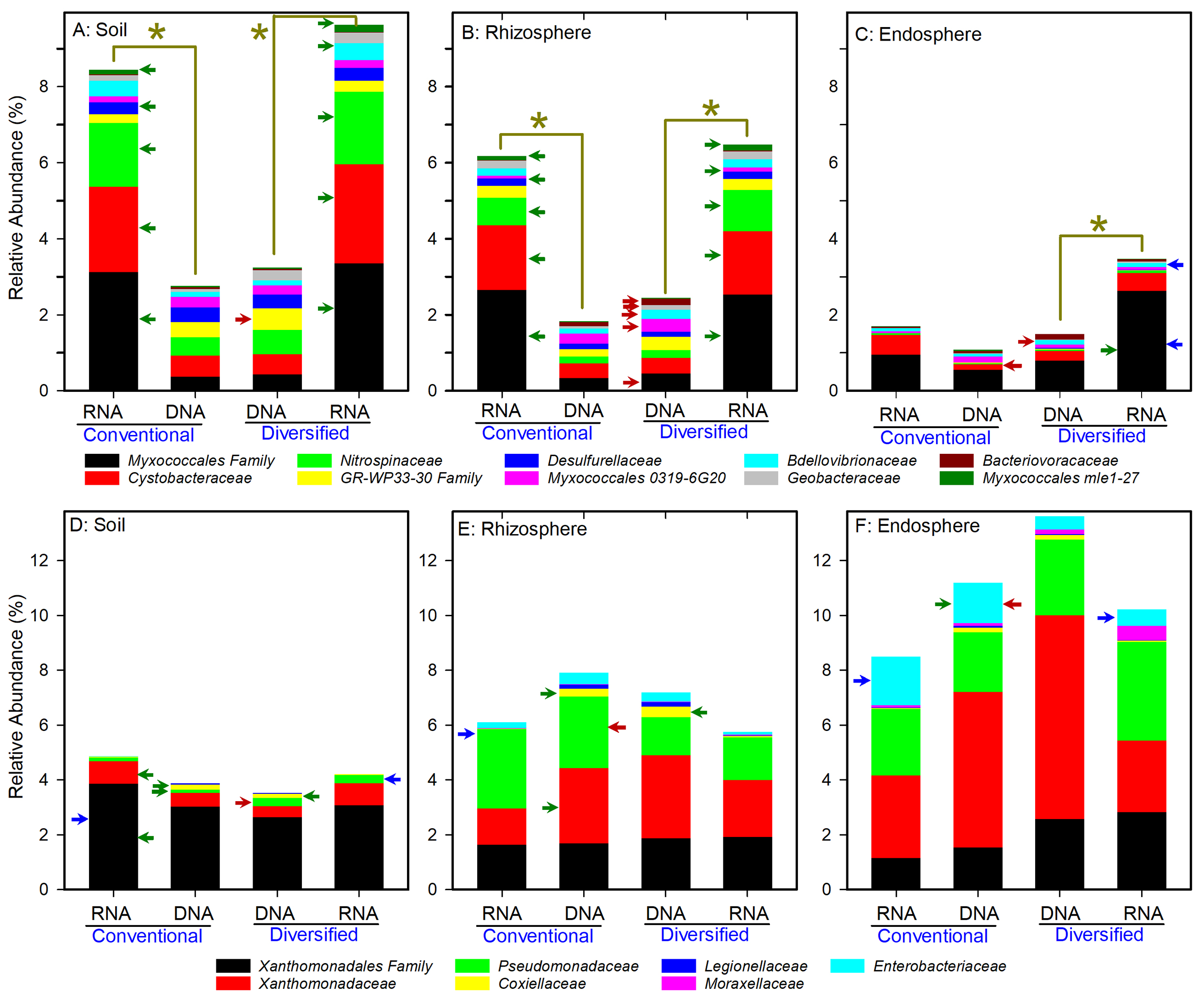

Supplement: FIG S1 [file msystems.00651-21-sf001.jpg]

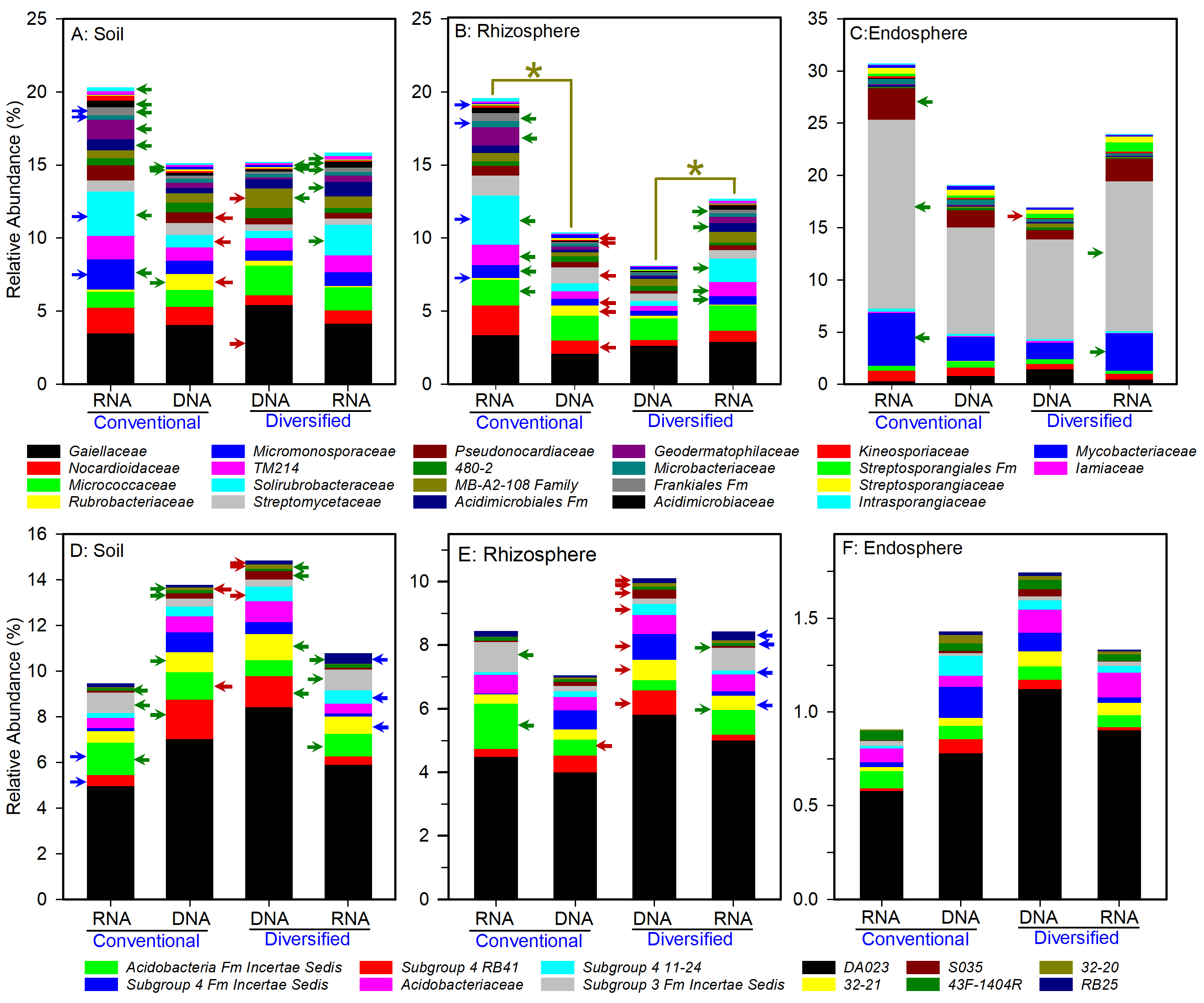

Supplement: FIG S2 [file msystems.00651-21-sf002.jpg]

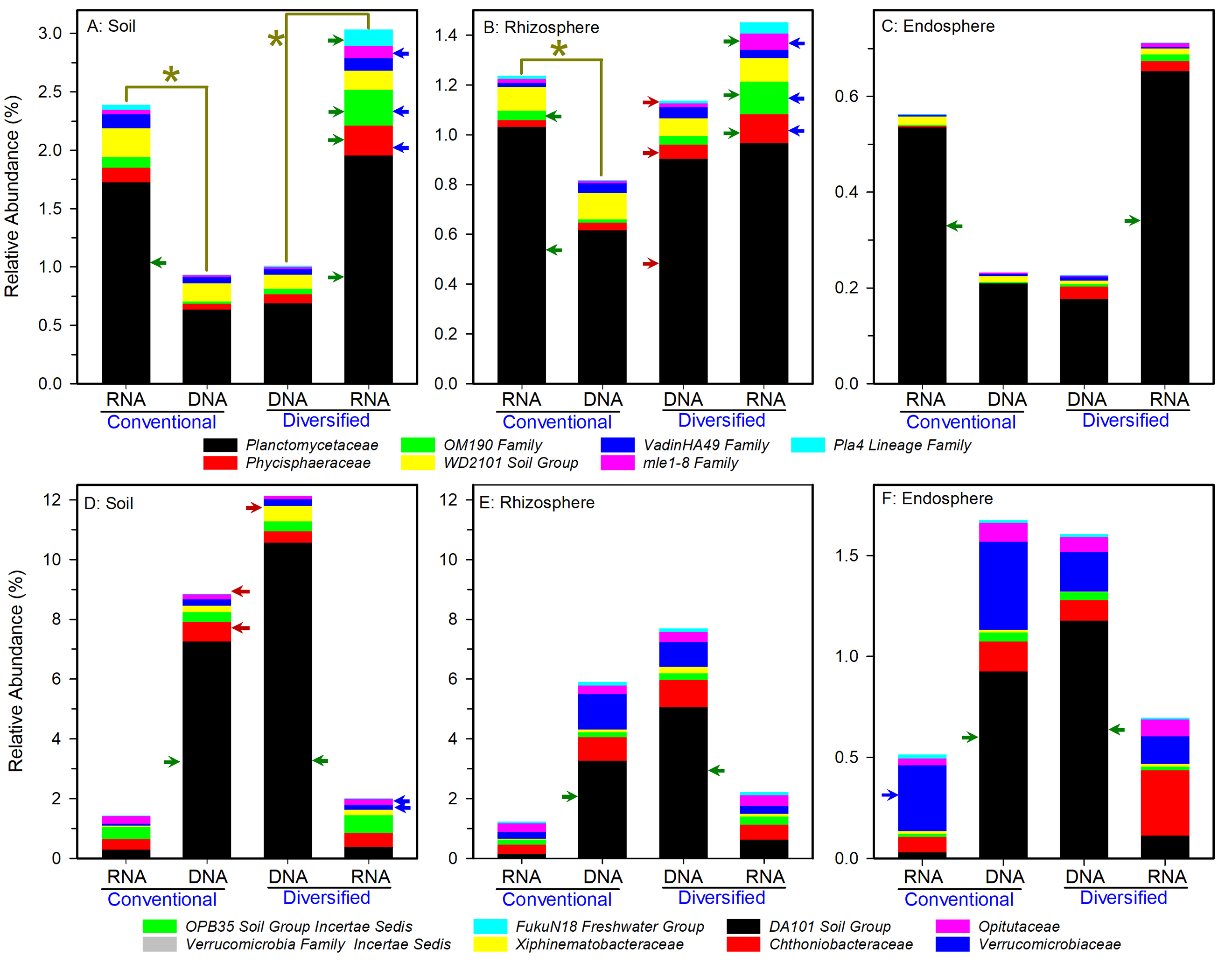

Supplement: FIG S3 [file msystems.00651-21-sf003.jpg]

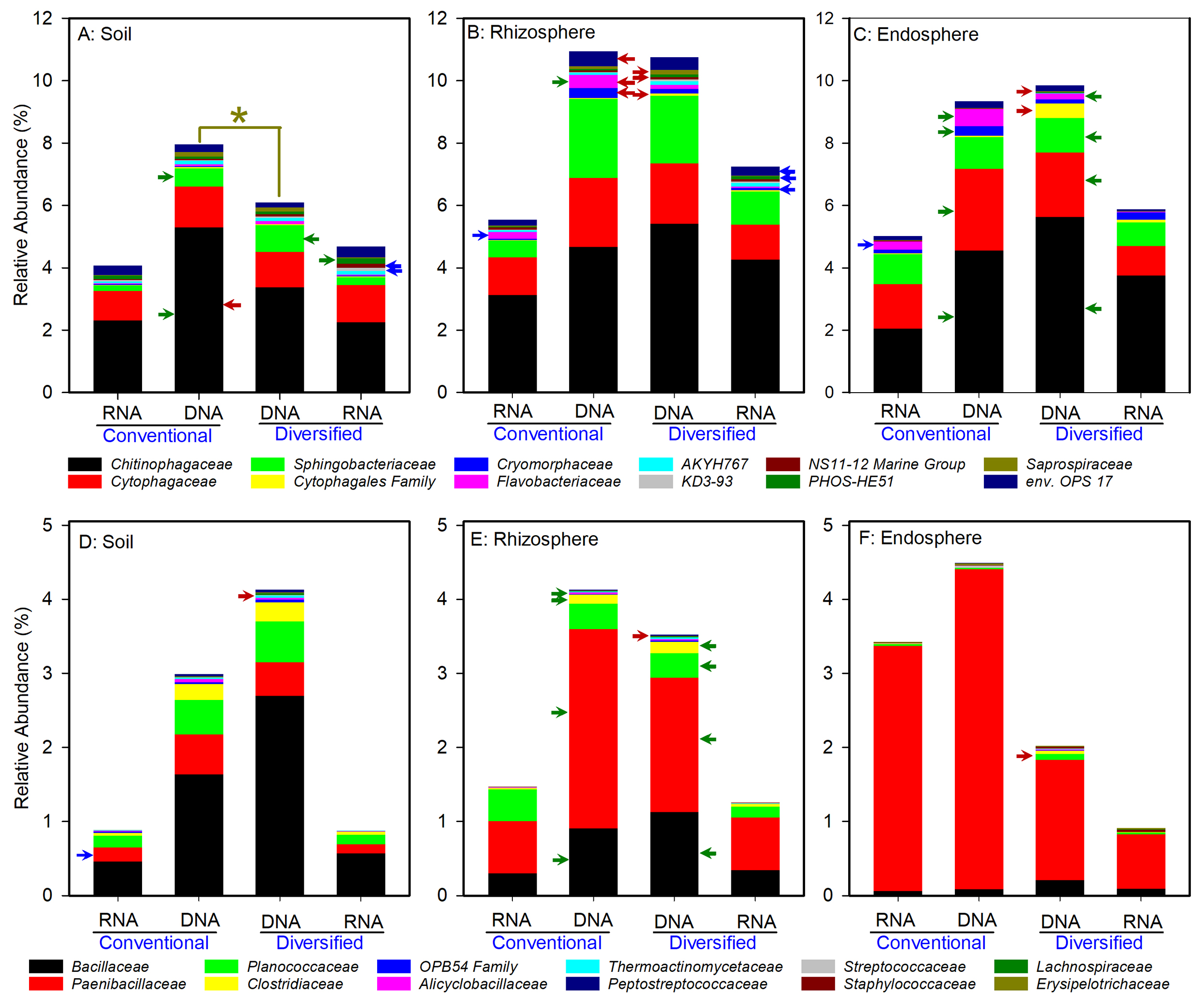

Supplement: FIG S4 [file msystems.00651-21-sf004.jpg]

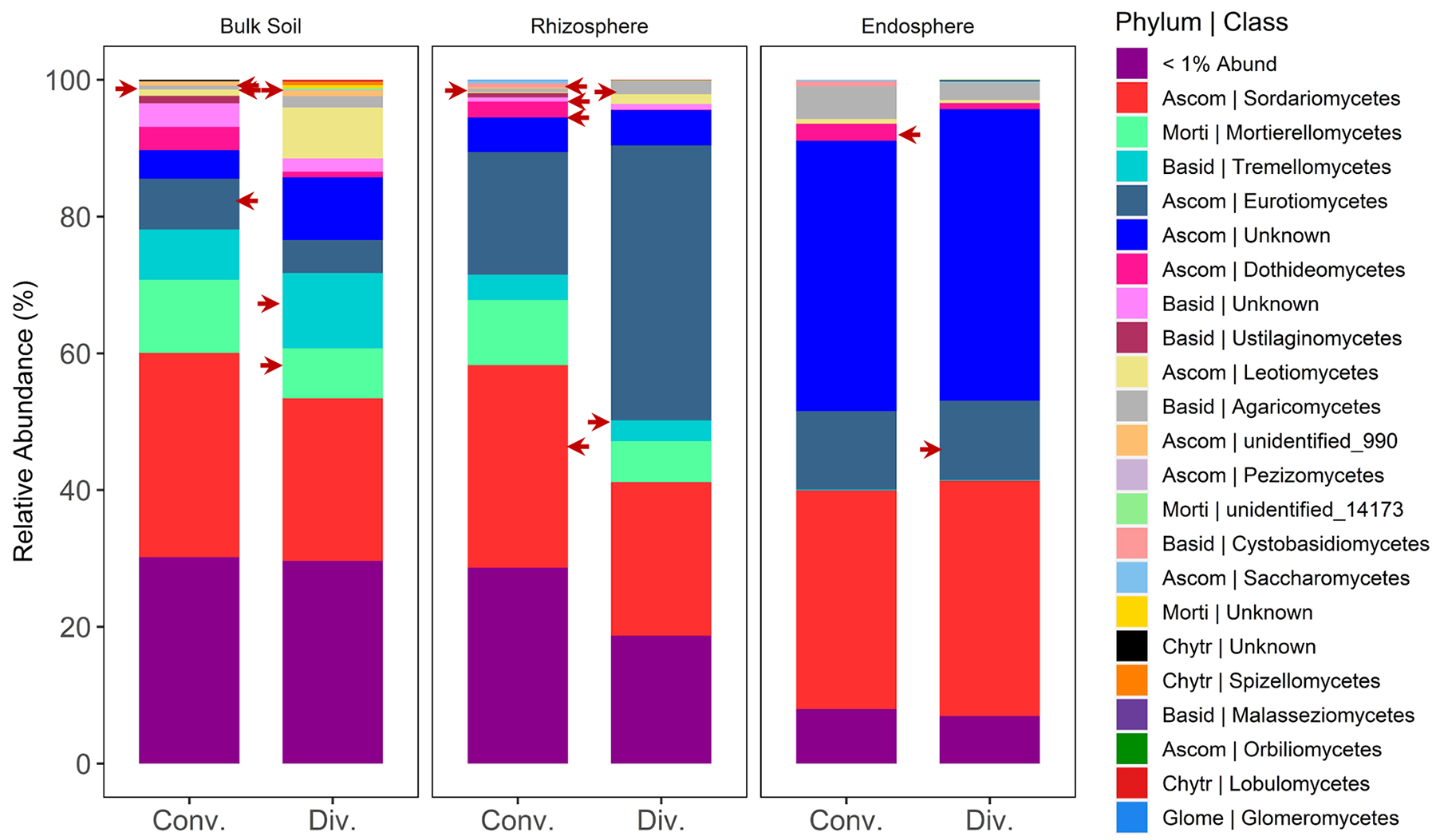

Supplement: FIG S5 [file msystems.00651-21-sf005.jpg]
