# Supplementary material for: Agricultural Management Affects the Active Rhizosphere Bacterial Community Composition and Nitrification
Source: mSystems. 2021 Sep 28;6(5):e00651-21. doi: 10.1128/mSystems.00651-21 (PMC8547420; doi:10.1128/mSystems.00651-21)
Supplement: TABLE S3 [file msystems.00651-21-st003.pdf]

Table S3 Analysis of variance of ammonia oxidizers and their potential metabolic activity.

	Nitrification Potential	Gross Nitrate Production	NO_3	AOB	AOA	AOA/AOB
Rhizosphere effect	0.020	<0.001	<0.001	<0.001	<0.001	<0.001
Cropping system	<0.001	0.739	0.011	<0.001	0.094	<0.001
Cropping system x Rhizosphere effect	<0.001	0.629	0.016	0.484	0.777	0.328

Separate 2-way ANOVAs were performed on Box-Cox transformed data for each trait. Values are *p*-values for each test.
