# Supplementary material for: Agricultural Management Affects the Active Rhizosphere Bacterial Community Composition and Nitrification
Source: mSystems. 2021 Sep 28;6(5):e00651-21. doi: 10.1128/mSystems.00651-21 (PMC8547420; doi:10.1128/mSystems.00651-21)
Supplement: TEXT S1 [file msystems.00651-21-s0001.pdf]

Supplemental Text S1 Detailed Materials and Methods

Experimental site description, soil collection and soil analyses. Soil was collected from Iowa State University's Marsden Long-term Cropping System Experiment (1), established in 2002 in Boone County, IA, USA (42°01' N, 93°78' W, 333 m above sea level), during the last week of June 2014. Soils, management practices, and site experimental design are described in Davis *et al*., 2012 (1). We sampled from two management regimes, a conventional system comprised of a two-year rotation of maize (*Zea mays*) and soybean (*Glycine max*) with inorganic N fertilization, and a diversified system comprised of a four-year rotation (maize, soybean, oat [*Avena sativa*]/alfalfa [*Medicago sativa*] and alfalfa) which received composted manure the fall preceding maize and, when warranted, a reduced inorganic N fertilizer side dress.

For each plot planted in corn in 2014 (i.e. 2 cropping systems × 4 blocks = 8 plots in total), five top-soil samples (0-20 cm, 0.2 m^2 each) were randomly and aseptically collected. Sampling occurred over the length of the plot approximately 2 m from plot boundaries to minimize edge effects. Soil was pooled by plot and stored in darkness at 20°C prior to sieving (4 mm); packing of rhizotrons occurred within 48 h of soil collection. Soil physicochemical properties are described in Table S1. Soil pH was determined with ~10 g dry weight in a 1:1 soil and water mixture. For elemental analysis subsamples of ~20 g wet weight were used. Total C/total N/organic matter levels analyzed by combustion, P/K/Cu/Fe contents by Mehlich-3 extraction, and NH_4^+/NO_3^- contents by colorimetry by the Soil & Plant Analysis Laboratory at Iowa State University, USA, using standard protocols.

Rhizotrons, growth conditions and sampling protocols. Rhizotrons are experimental root box systems [19 × 40 × 5 cm, modified after (2, 3)], which allow rapid and non-destructive access to the entire root system of a plant, as well as to soil in direct contact with these roots. For each

cropping system, two rhizotrons were filled with soil from each block (i.e. 2 cropping systems × 4 blocks × 2 replicates = 16 rhizotrons in total), to a bulk density of 1.1 g soil cm^{-3}, corresponding to the average bulk density of the upper soil layer at the experimental site. The soil was maintained at a 65% water-holding capacity and watered when necessary with sterile deionized water. Watered rhizotrons were allowed to equilibrate for 24 h prior to planting two maize seedlings (Viking 72-04N, Albert Lea Seed House, MN, USA) in each rhizotron, giving a total of four plants per plot × cropping system combination (= 32 plants total). Seeds were surface-sterilized with a 6% sodium hypochlorite solution for 10 min, followed by several rinses with sterile deionized water and then pre-germinated in the dark on filter paper in sterile Petri dishes moistened with 7.5 mL of a 2 g L^{-1} Captan (Bonide Products, Inc., NY, USA), for 60 hours at 25°C. Rhizotrons were maintained at 24°C and a 16/8 hours light/dark photoperiod (350 µmol photons m^{-2} s^{-1}). Harvesting occurred 21 days after planting, corresponding to vegetative stage V4/V5.

Rhizotrons were disassembled in sterile plastic bins to expose roots and to aseptically collect soil samples (~1 g) at least 1 cm from a root. The entire root system of a plant was then extracted and the remaining bulk soil removed by gentle shaking. The rhizosphere, defined as the fine soil adhering to roots, was obtained by immersing the whole root system in ice-cold 0.1 M phosphate buffer and vortexing briefly [as described previously (4)]. Roots were transferred to fresh phosphate buffer and sonicated for 1.5 min at 20 W using a needle probe. Washates and sonicates were pooled prior to filtering (100-µm nylon mesh) to remove soil particles and plant debris. This filtrate was centrifuged for 5 min at 4°C and 14,000 x g (LegendRT+, Fiberlite F-15 rotor; Thermo Fischer Scientific Inc., MA, USA). Pellets were then resuspended in 1.5 mL of sterile deionized water and centrifuged for 1 min, as described above, and the supernatant was decanted to obtain a rhizosphere sample. Prior to obtaining endosphere samples, soil-free roots were transferred to 40

mL ice-cold autoclaved NanoPure water and sonicated for 1.5 min at 20 W, prior to decanting the sonicate. This sonication step was repeated two more times prior to scanning roots on a flatbed scanner for automatic root image analysis (5). Roots were then freeze-dried and weighed prior to pulverization for endosphere sampling. All samples were kept at -80°C.

DNA and RNA extractions. DNA was extracted from soil and rhizosphere samples with the PowerSoil Kit, and the endophytic samples with the PowerPlant Pro Kit (MoBio Laboratories, CA, USA) per manufacturer's instructions, with a Powerlyzer 24 homogenizer (2,500 rpm, 30 s, 24°C). The DNA concentrations were determined using a Nanodrop 1000 spectrophotometer (Thermo Fischer Scientific Inc., MA, USA). RNA was obtained using the PowerSoil Total RNA Kit (MoBio Laboratories, CA, USA) per manufacturer's instructions, followed by DNase treatment (TURBO DNA-*free* Kit, Life Technologies, CA, USA) and first and second strand cDNA synthesis (NEBNext RNA First Strand and mRNA Second Strand Synthesis Modules, New England BioLabs, MA, USA). The quality of the cDNA was assessed with a 2100 Bioanalyzer. All samples were stored at -80°C.

Amplicon sequencing and processing. For the 16S rRNA V4 region, DNA samples were sent to Argonne National Laboratory (Lemont, IL, USA) for amplicon library preparation and sequencing using the 515/806R primers (6). For the fungal ITS region, DNA was sequenced at the University of Minnesota Genome Center using the ITS1/ITS2 primers. Amplicons of 16S rRNA and ITS were paired-end sequenced (250 bp) on Illumina MiSeq instruments in separate runs. Three replicates of genomic DNA from Microbial Mock Community B (BEI Resources, kindly provided by Dr. Sarah Highlander) or three replicates of fungal mock community DNA (7) were included with the 16S rRNA and ITS libraries.

For the 16S rRNA sequences, we only used the forward sequence since the reverse sequence was of insufficient quality. We used Cutadapt (8) to remove the primer and adapter sequences, Trimmomatic (9) to trim and quality-filter reads (Q25), and QIIME to demultiplex the samples (10). Chloroplast and mitochondrial reads were removed with Cutadapt (8). Following clustering of OTUs with UPARSE (11), singletons and chimeras were filtered out. The outputs from UPARSE were used to produce an OTU table in BIOM format. A total of 5,453,802 reads were generated, with a median sequence length of 253 bp. We assigned taxonomy to the OTU table using SILVA [version 118 (12)] and used PyNAST (13) to align all of the 16S rRNA sequences before filtering out unaligned OTUs, and a phylogenetic tree was then produced using "make_phylogeny.py" in QIIME.

For fungal ITS, we only used the forward sequences since the reverse reads were of insufficient quality. Trimmomatic was also used to remove primers and for sliding-window quality trimming at Q25. DADA2 version 1.10.1 (14) was used to filter out phiX DNA and to dereplicate sequences, and to generate a parametric error model for sample inference. An OTU table was generated and chimeras removed in DADA2 using default parameters. Taxonomy was assigned using UNITE [version 8.0 (15)]. All sequences were deposited to the NCBI Sequence Read Archive (PRJNA686799 and PRJNA685216).

Data analysis. Distance matrices of 16S rRNA OTUs were created in QIIME using weighted and unweighted UniFrac distances (16) and Bray-Curtis dissimilarity while ITS OTUs were examined using Bray-Curtis dissimilarity distances. A comparison between rarefied and non-rarefied data revealed similar trends in β-and α-diversity metrics, and thus subsequent analyses were performed on non-rarefied data (17). Further analyses were carried out using the PHYLOSEQ package (18), and LEfSe [Linear Discriminant Analysis Effect Size (19)] was used

to identify differentially-abundant taxa at the family and OTU levels (α-value for Kruskal-Wallis test among classes and for pairwise Wilcoxon test between subclasses = 0.05, threshold on the logarithmic LDA score for discriminative features = 2.0, strategy for multi-class analysis = all-against-all [more strict]).

Mock bacterial communities were analyzed by averaging the triplicate counts for each OTU, and we calculated the ratio of that member relative to the entire community at various taxonomic ranks and compared that to the expected ratio as defined in the mock community (Table S4). Because only 3 out of the 17 members were under-represented at the genus level, Family-level resolution was used for identifying differentially-abundant taxa, while OTU-level resolution was used to quantify the total number of differentially-abundant taxa.

Assessment of ammonia-oxidizer abundance. The *amoA* gene primer sets for AOB and AOA were *amoA*-1F/2R (20) and Arch-*amoA*F/R (21), respectively, in a 20 µl mixture containing 1X PerfeCTa SYBR Green FastMix (Quanta BioSciences Inc., MD, USA), 0.3 µM of each primer, 0.0015 mg/µl BSA (for AOB only), and 20 ng DNA (5 µl). The PCR conditions were 95°C for 3 min, followed by 40 cycles of 30 s at 95°C, 60 s at 55°C, 45 s at 72°C, and a final step of 5 min at 72°C. The qPCR standards consisted of a 1:1:1 mixture of known concentrations of 3 synthesized *amoA* fragments (G-blocks, IDT, IA, USA), the same length as our target amplicons, each with 0-2 mismatches with the primers. Derivation of the gBlocks was previously described (22) and standard curves were generated from a six-point, 10-fold dilution series of either AOB or AOA gBlocks mixtures. Each reaction was performed in triplicate in a Realplex^2 Mastercycler (Eppendorf, Hamburg, Germany). Product specificity was confirmed by melting curve analysis and agarose gel visualization. Amplification efficiencies were approximately 92% for both AOB and AOA ($R^2 > 0.996$ for both).

Nitrification potential and gross nitrate production. For determining the nitrification potential and gross nitrate production rates, separate rhizotrons with and without maize plants were prepared. Nitrification potential was determined as described previously (23). For determining gross NO_3 production rates, we removed the front and back faces of the rhizotron to expose the soil. We then sufficiently wetted each face of the block of soil by spraying 100 mL of a 30% APE $^{15}KNO_3$ solution. We conducted two experiments, where in the first 10 to 15 g of soil was randomly collected (avoiding root-free areas) while in the second the soil was homogenized before collecting subsamples after 15-min, 3-h and 24-h incubation. Statistical analyses indicated there was no significant experiment effect (data not shown), and thus results from both experiments were combined. Soil samples were immediately subjected to an ice-cold KCl 2M extraction (100 mL), for $^{15}N/^{14}N$ ratio determination at Utah State University, as described previously (24, 25). To determine the amount of $^{15}NO_3$ to apply, we measured inorganic N pool sizes in five rhizotrons with and without plants at the ISU Soil & Plant Analysis Laboratory. Subsamples of bulk and rhizosphere soils (~10 g) were also collected for measuring soil physicochemical properties.

Statistical analyses. Amplicon data was analyzed with non-parametric permutational multivariate analyses of variance (PERMANOVA) using distance matrices (ADONIS, 999 permutations) (26). PERMANOVAs and elemental and root trait ANOVAs were performed in R version 3.1.1 on Box-Cox- or square-root-transformed data when appropriate (27-29). Separate two-factor ANOVAs of AOA and AOB qPCR-based abundance estimates, nitrification potentials, nitrate pool sizes and gross nitrate production rates were analyzed on Box-Cox-transformed data using JMP13 (SAS Institute, Cary, NC, USA). Post-hoc tests were determined by Tukey's HSD when comparisons were of interest. Species diversity was analyzed using JMP13; richness indices

(total OTUs and Chao1) were tested by ANOVAs, with post-hoc treatment comparisons based on Fisher's LSD, while the Simpson index was examined by Kruskal-Wallis tests.

REFERENCES

1. Davis AS, Hill JD, Chase CA, Johanns AM, Liebman M. 2012. Increasing cropping system diversity balances productivity, profitability and environmental health. PLoS ONE 7:e47149.
2. DeAngelis KM, Brodie EL, DeSantis TZ, Andersen GL, Lindow SE, Firestone MK. 2009. Selective progressive response of soil microbial community to wild oat roots. ISME J 3:168-78.
3. Jaeger CH, 3rd, Lindow SE, Miller W, Clark E, Firestone MK. 1999. Mapping of sugar and amino acid availability in soil around roots with bacterial sensors of sucrose and tryptophan. Appl Environ Microbiol 65:2685-90.
4. Wattenburger CJ, Halverson LJ, Hofmockel KS. 2019. Agricultural management affects root-associated microbiome recruitment over maize development. Phytobiomes Journal 3:260-272.
5. Pace J, Lee N, Naik HS, Ganapathysubramanian B, Lubberstedt T. 2014. Analysis of maize (Zea mays L.) seedling roots with the high-throughput image analysis tool ARIA (Automatic Root Image Analysis). PLoS One 9:e108255.
6. Caporaso JG, Lauber CL, Walters WA, Berg-Lyons D, Huntley J, Fierer N, Owens SM, Betley J, Fraser L, Bauer M, Gormley N, Gilbert JA, Smith G, Knight R. 2012. Ultra-high-

throughput microbial community analysis on the Illumina HiSeq and MiSeq platforms. ISME J 6:1621-1624.

7. Bakker MG. 2018. A fungal mock community control for amplicon sequencing experiments. Molecular Ecology Resources 18:541-556.
8. Martin M. 2011. Cutadapt removes adapter sequences from high-throughput sequencing reads. EMBnetjournal doi:10.14806/ej.17.1.200.
9. Bolger AM, Lohse M, Usadel B. 2014. Trimmomatic: A flexible trimmer for Illumina sequence data. Bioinformatics doi:10.1093/bioinformatics/btu170.
10. Caporaso JG, Kuczynski J, Stombaugh J, Bittinger K, Bushman FD, Costello EK, Fierer N, Pena AG, Goodrich JK, Gordon JI, Huttley GA, Kelley ST, Knights D, Koenig JE, Ley RE, Lozupone CA, McDonald D, Muegge BD, Pirrung M, Reeder J, Sevinsky JR, Turnbaugh PJ, Walters WA, Widmann J, Yatsunenko T, Zaneveld J, Knight R. 2010. QIIME allows analysis of high-throughput community sequencing data. Nat Methods 7:335-6.
11. Edgar RC. 2013. UPARSE: highly accurate OTU sequences from microbial amplicon reads. Nature Methods 10:996.
12. Quast C, Pruesse E, Yilmaz P, Gerken J, Schweer T, Yarza P, Peplies J, Glöckner FO. 2013. The SILVA ribosomal RNA gene database project: Improved data processing and web-based tools. Nucleic Acids Research doi:10.1093/nar/gks1219.
13. Caporaso JG, Bittinger K, Bushman FD, Desantis TZ, Andersen GL, Knight R. 2010. PyNAST: A flexible tool for aligning sequences to a template alignment. Bioinformatics doi:10.1093/bioinformatics/btp636.

14. Callahan BJ, McMurdie PJ, Rosen MJ, Han AW, Johnson AJA, Holmes SP. 2016. DADA2: High-resolution sample inference from Illumina amplicon data. Nature Methods 13:581-583.

15. Nilsson RH, Larsson KH, Taylor AFS, Bengtsson-Palme J, Jeppesen TS, Schigel D, Kennedy P, Picard K, Glöckner FO, Tedersoo L, Saar I, Kõljalg U, Abarenkov K. 2019. The UNITE database for molecular identification of fungi: Handling dark taxa and parallel taxonomic classifications. Nucleic Acids Research doi:10.1093/nar/gky1022.

16. Lozupone C, Knight R. 2005. UniFrac: a new phylogenetic method for comparing microbial communities. Appl Environ Microbiol 71:8228-35.

17. McMurdie PJ, Holmes S. 2014. Waste not, want not: Why rarefying microbiome data is inadmissible. PLoS Comput Biol 10:e1003531.

18. McMurdie PJ, Holmes S. 2013. phyloseq: an R package for reproducible interactive analysis and graphics of microbiome census data. PLoS One 8:e61217.

19. Segata N, Izard J, Waldron L, Gevers D, Miropolsky L, Garrett WS, Huttenhower C. 2011. Metagenomic biomarker discovery and explanation. Genome Biology 12.

20. Rotthauwe JH, Witzel KP, Liesack W. 1997. The ammonia monooxygenase structural gene amoA as a functional marker: molecular fine-scale analysis of natural ammonia-oxidizing populations. Appl Environ Microbiol 63:4704-12.

21. Francis CA, Roberts KJ, Beman JM, Santoro AE, Oakley BB. 2005. Ubiquity and diversity of ammonia-oxidizing archaea in water columns and sediments of the ocean. Proc Natl Acad Sci U S A 102:14683-14688.

22. Wattenburger CJ, Gutknecht J, Zhang Q, Brutnell TP, Hofmockel KH, Halverson LJ. 2020. The rhizosphere and cropping system, but not arbuscular mycorrhizae, affect ammonia

oxidizing archaea and bacteria abundances in two agricultural soils. Applied Soil Ecology 151.

23. Herman DJ, Halverson LJ, Firestone MK. 2003. Nitrogen dynamics in an annual grassland: oak canopy, climate, and microbial population effects. Ecological Applications 13:593-604.
24. Hart SC, Stark JM, Davidson EA, Firestone MK. 1994. Nitrogen mineralization, immobilization, and nitrification, p 985-1018. *In* al. RWWe (ed), Methods of Soil Analysis. Soil Science Society of America, Madison.
25. Herman DJ, Johnson KK, Jaeger CH, III, Schwartz E, Firestone MK. 2006. Root Influence on Nitrogen Mineralization and Nitrification in Avena barbata rhizosphere soil. Soil Sci Soc Am J 70:1504-1511.
26. Dixon P. 2003. VEGAN, a package of R functions for community ecology doi:10.1111/j.1654-1103.2003.tb02228.x.
27. Osborne JW. 2010. Improving your data transformations: Applying the Box-Cox transformation. Practical Assessment, Research and Evaluation.
28. Box GEP, Cox DR. 1964. An analysis of transformations: Applying the Box-Cox transformation. Journal of the Royal Satistical Society.
29. Wessa P. 2015. Box-Cox Normality Plot (v1.1.11) in Free Statistics Software (v1.1.23-r7).
