# Supplementary material for: Agricultural Management Affects the Active Rhizosphere Bacterial Community Composition and Nitrification
Source: mSystems. 2021 Sep 28;6(5):e00651-21. doi: 10.1128/mSystems.00651-21 (PMC8547420; doi:10.1128/mSystems.00651-21)
Supplement: TABLE S4 [file msystems.00651-21-st004.pdf]

Table S4 Mock community identification at the genus level.

Prokaryote			Fungal		
	Percent			Percent	
Taxon	Expected	Actual	Taxon	Expected	Actual
Acinetobacter	0.22	0.37	*Alternaria*	0.03	ND
Actinomyces	0.02	0.01	*Aspergillis*	0.26	0.48
Bacillus	2.19	4.66	*Candida*	0.26	ND
Bacteroides	0.02	0.05	*Chytriomyces*	0.26	ND
Clostridium	2.19	2.44	*Claviceps*	0.13	ND
Deinococcus	0.02	0.01	*Fusarium*	29.12	38.82
Enterococcus	0.02	0.01	*Mortierella*	0.26	0.002
Escherichia-Shigella	21.91	25.00	*Filobasidiaceae*	13.11	0.003
Helicobacter	0.22	0.84	*Trichocomaceae*	0.52	0.003
Lactobacillus	0.22	0.12	*Rhizoctania*	0.26	0.04
Listeria	0.22	0.41	*Rhizomucor*	0.13	ND
Neisseria	0.22	0.48	*Rhizophagus*	0.026	ND
Propionibacterium	0.22	0.00	*Saccharomyces*	52.47	ND
Pseudomonas	2.19	0.01	*Saitoella*	2.62	ND
Rhodobacter	21.91	0.36	*Trichoderma*	0.26	0.002
Staphylococcus	24.1	34.4	*Ustilago*	0.26	ND
Streptococcus	24.12	29.01			
